# The efficacy and safety of autologous epidermal cell suspensions for re‐epithelialization of skin lesions: A systematic review and meta‐analysis of randomized trials

**DOI:** 10.1111/srt.13820

**Published:** 2024-06-19

**Authors:** Jiaqi Lou, Ziyi Xiang, Youfen Fan, Jingyao Song, Neng Huang, Jiliang Li, Guoying Jin, Shengyong Cui

**Affiliations:** ^1^ Burn Department Ningbo No. 2 Hospital Ningbo Zhejiang Province China; ^2^ Section of Medical Psychology Faculty of Medicine Department of Psychiatry and Psychotherapy University of Bonn Bonn Germany; ^3^ School of Mental Health Wenzhou Medical University Whenzhou Zhejiang Province China

**Keywords:** autologous epidermal cell suspensions, complications, healing time, meta‐analysis, repigmentation, skin lesion, ulcer, vitiligo, wound

## Abstract

**Background:**

Successful usage of autologous skin cell suspension (ASCS) has been demonstrated in some clinical trials. However, its efficacy and safety have not been verified. This latest systematic review and meta‐analysis aim to examine the effects of autologous epidermal cell suspensions in re‐epithelialization of skin lesions.

**Methods:**

Relevant articles were retrieved from PubMed, Embase, Cochrane Database, Web of Science, International Clinical Trials Registry Platform, China National Knowledge Infrastructureris, VIP Database for Chinese Technical Periodicals and Wanfang database. The primary output measure was the healing time, and the secondary outputs were effective rate, size of donor site for treatment, size of study treatment area, operation time, pain scores, repigmentation, complications, scar scale scores and satisfaction scores. Data were pooled and expressed as relative risk (RR), mean difference (MD) and standardized mean difference (SMD) with a 95% confidence interval (CI).

**Results:**

Thirty‐one studies were included in this systematic review and meta‐analysis, with 914 patients who received autologous epidermal cell suspensions (treatment group) and 883 patients who received standard care or placebo (control group). The pooled data from all included studies demonstrated that the treatment group has significantly reduced healing time (SMD = −0.86; 95% CI: −1.59–0.14; *p* = 0.02, I^2 ^= 95%), size of donar site for treatment (MD = −115.41; 95% CI: −128.74–102.09; *p*<0.001, I^2 ^= 89%), operation time (MD = 25.35; 95% CI: 23.42–27.29; *p*<0.001, I^2 ^= 100%), pain scores (SMD = −1.88; 95% CI: −2.86–0.90; *p* = 0.0002, I^2 ^= 89%) and complications (RR = 0.59; 95% CI: 0.36–0.96; *p* = 0.03, *I* ^2 ^= 66%), as well as significantly increased effective rate (RR = 1.20; 95% CI: 1.01–1.42; *p* = 0.04, *I* ^2 ^= 77%). There were no significant differences in the size of study treatment area, repigmentation, scar scale scores and satisfaction scores between the two groups.

**Conclusion:**

Our meta‐analysis showed that autologous epidermal cell suspensions is beneficial for re‐epithelialization of skin lesions as they significantly reduce the healing time, size of donar site for treatment, operation time, pain scores and complications, as well as increased effective rate. However, this intervention has minimal impact on size of treatment area, repigmentation, scar scale scores and satisfaction scores.

**Abbreviations**
ASCS: autologous epidermal cell suspensions
CI: confidence interval
ECS: epidermal cell suspension
ECS: epidermal cell suspension
EMT: epidermal melanocyte transfer
HFCS: hair follicle cell suspension
HFMT: hair follicular melanocyte transfer
MD: mean difference
NCES: autologous non‐cultured epidermal cell suspension
NCORSHFS: autologous non‐cultured outer root sheath hair follicle cell suspension
PRFM: platelet‐rich fibrin matrix
RCTs: randomized controlled trial
RR: relative risk
SBEG: suction blister epidermal grafting
SC: standard Care
SMD: standardized mean difference
SSG: smash skin grafting
STSG: split‐thickness skin grafting

## 1 BACKGROUND

With the progression of daily life, production, transportation, as well as the development of radiation and chemotherapy, chronic illnesses, and an aging population,^1^ the skin being the outermost physical defense barrier of the human body, it is often the first to suffer damage. Common skin disorders like trauma, burns, pigment abnormalities, scars, and chronic ulcers pose significant physiological distress, mental strain, and economic burden to patients.^2^, ^3^ One of the shared challenges among fields such as burn treatment, aesthetics, and reconstructive repair is restoring the normal form and functionality of damaged skin. Re‐epithelization of wounds is a crucial phase in healing processes. In cases of burns, trauma, and medically‐induced injuries, a deficit of skin islands on wound surfaces may prevent immediate healing.^4^ An optimal treatment strategy would consist of transplanting matching epidermal cells to the affected area and fostering their growth within suitable environments to expedite the re‐epithelization process, improving wound repair quality, and averting scar formation.^5^ Considering the principle of skin injury healing, a variety of treatment methods have been employed in clinical settings, aiming to counter different types of skin injuries. These include autologous skin grafting for burn treatment, negative pressure wound therapy (NPWT) for addressing various types of acute and chronic ulcers, blister epidermal grafting for treating pigment anomalies (like vitiligo), or laser therapy used for scar treatment.^6^, ^7^ These therapeutic approaches vary in effectiveness, and sometimes, a combination is required for skin damage repair. Current studies are exploring the optimal method to repair skin injuries.

With advancements in medical technology, a novel technique—Autologous Skin Cell Suspensions (ASCSs) has been extensively applied to burns, aesthetics, and plastic surgery areas for skin wound repair, yielding notable results.^8^ ASCS builds upon the existing autologous epidermal cell grafting method, employing particular technology for autologous skin cell collection, processing, and transplantation.^9^ This involves converting autologous skin cells into suspension containing various cell components like keratinocytes and melanocytes and spraying them onto wounds for repair. The ReCell technology^10^ extends this approach, preparing the necessary epidermal cell suspension for wound repair in vitro without the need for cell culture, falling under the Autologous Non‐Cultured Epidermal Cell Suspension (NCES) technique.^11^ Compared to traditional treatment methods like skin grafting, research found that autologous epidermal cell suspensions have several advantages including alleviation of post‐operative pain, reduction of post‐operative scars, shorter hospitalization duration, restoration of original skin color, and cost reduction, while requiring a smaller donor site area.^12^, ^13^ Despite the increasing number of randomized controlled trials (RCTs)^14^ addressing the treatment effects of autologous epidermal cell suspensions on various skin damages, due to the insufficient number of studies and design flaws in RCTs, the efficacy and safety of autologous epidermal cell suspensions in skin damage repair are yet to be determined. To this end, this systematic review and meta‐analysis aims to assess the efficacy and safety of autologous epidermal cell suspensions for re‐epithelialization of skin lesions.

## 2 METHODS

The research protocol, outcomes, and relevant items in this systematic review were reported in accordance with the Preferred Reporting Items for Systematic Reviews and Meta‐analyses (PRISMA) Statement.^15^ The protocol for this meta‐analysis has been registered with PROSPERO (CRD42024507178).

### 2.1 Data source and search strategy

Relevant articles were searched in PubMed, Embase, Cochrane Database, Web of Science, International Clinical Trials Registry Platform, China National Knowledge Infrastructureris, VIP Database for Chinese Technical Periodicals and Wanfang database using subject headings and keywords containing “Skin disease”, “Skin and Connective Tissue Diseases”, “Burns”, “Epidermis”, “Dermis”, “Hair Follicle”, “Sweat Glands”, “Sebaceous Glands”, “Apocrine Glands”, “Eccrine Glands”, “Wounds and Injuries”, “Trauma”, “Ulcers”, “Vitiligo”, “Hypopigmentation” and “Pigmentation Disorders”. In addition, the references of the included studies and relevant review articles were screened to identify eligible clinical trials that were not found through the database searches. The identified articles were imported, stored, and managed by EndNote 20. Each search result was independently reviewed for eligibility by two authors (Jiaqi Lou and Ziyi Xiang), and any disagreement was resolved by the corresponding author (Shengyong Cui).

### 2.2 Eligibility criteria

Inclusion criteria: (1) Type of study: RCT; (2) Participants: Patients who had skin lesions and were hospitalized of all ages; (3) Intervention: autologous epidermal cell suspensions alone or in combination with other treatments compared with standard care or placebo; and (4) Outcomes: The healing time, effective rate, size of donor site for treatment, size of study treatment area, operation time, pain scores, repigmentation, complications, scar scale scores and satisfaction scores.

Exclusion criteria: (1) Studies without clear inclusion; (2) Outcomes that had not been clearly stated; (3) Uncontrolled studies; (4) Preclinical studies in animal models.

If multiple articles reported the same or overlapping data, the article with the longer duration of the intervention or larger sample size was included in this study.

### 2.3 Data extraction and quality assessment

Articles were independently selected by two co‐authors (Jiaqi Lou and Ziyi Xiang) by screening the titles and/or abstracts, and full‐text articles were then evaluated for eligibility and adequacy in information. For each study that fulfilled the eligibility criteria, study sources (author, publication year, journal and country), characteristics of study population (types of skin lesions, skin lesion area, sample size, study design, type of subjects, male to female ratio, baseline mean age, duration of follow‐up, pain score, scar scale scores and satisfaction scores), characteristics of intervention, and outcomes (including at least one of the following: The healing time, effective rate, size of donor site for treatment, size of study treatment area, operation time, pain scores, repigmentation, complications, scar scale scores and satisfaction scores were extracted using a standard data extraction template and the Microsoft Excel (Version number: 16.78.3).

The methodological quality of each included RCT was assessed using the Cochrane Collaboration's risk of bias tool^16^ based on the Cochrane Handbook for Systematic Reviews of Interventions Version 5.0.1. The risk of bias for each item was judged as low, high, or unclear based on the criteria of the Cochrane Handbook for Systematic Reviews.^17^ Data extraction and quality assessment were performed independently by two authors (Jiaqi Lou and Shengyong Cui), and any discrepancy was settled by discussion and consensus. When a consensus was not reached, a third researcher (Youfen Fan) acted as an arbitrator. Data synthesis was performed using Review Manager (RevMan) 5.4 by another author (Jingyao Song).

### 2.4 Statistical analysis

**FIGURE 1:**
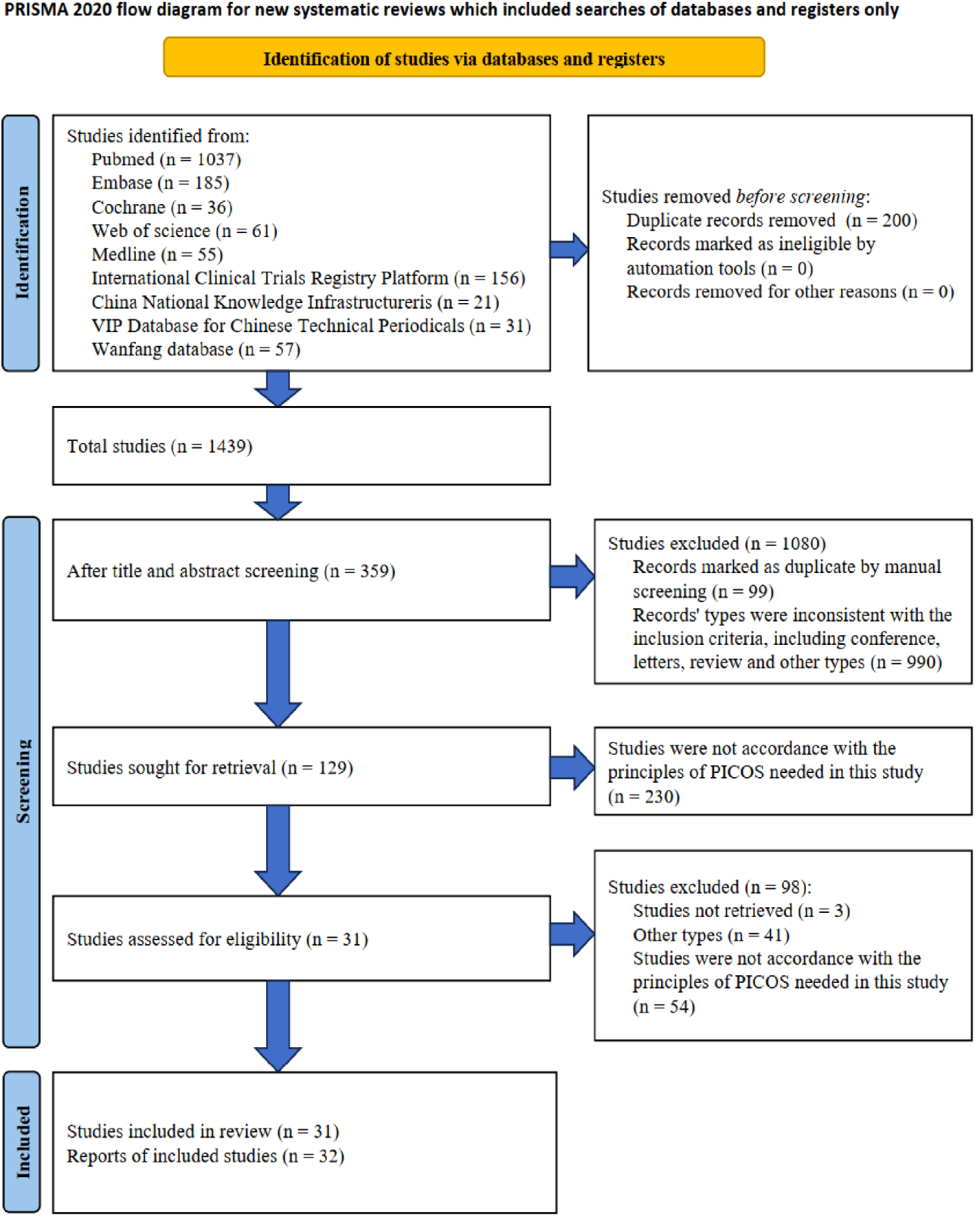
PRISMA diagram detailing the literature search and the study selection/exclusion process. PRISMA, preferred reporting items for systematic reviews and meta‐analyses; RCT, randomized controlled trials.

Data were extracted independently by two authors (Jiaqi Lou and Jiliang Li) from the full texts of the studies and compiled into shared sheets. The following information was collected from the included studies: first author, year of publication, intervention and details of the outcome. Data were validated by a third author (Neng Huang) using a standardized method. The methodological quality assessment shown in Figure 1. was based on the Cochrane Reviewers’ Handbook.^16^, ^17^ Discrete numerical variables were reported in risk ratio (RR) and 95% confidence intervals (CIs). Continuous variables are expressed as standardized mean difference (SMD) and mean difference (MD). The SMD was used as a summary statistic in this meta‐analysis due to the application of different methods to assess the same outcome. The *I* ^2^ statistic was used to quantify heterogeneity, and forest plots were generated and double‐checked by two authors (Jiaqi Lou and Guoying Jin). If *I* ^2^ < 50%, the pooled outcomes were considered to have low statistical heterogeneity, and if *I* ^2^ > 50%, the pooled outcomes were considered to have high statistical heterogeneity.^12^ Each low heterogeneity analysis was estimated using a fixed‐effects model, otherwise, the random‐effects model was applied. The possible sources of heterogeneity were analyzed using one or more of the following methods^18^: (i) Subgroup analyses based on the characteristics of different percentage of repigmentation and different types of complications; and (ii) The potential publication bias in each analysis was assessed using funnel plots, in which *p* < 0.05 was considered statistically significant. All statistical analyses were performed using Revman 5.4. A *p* < 0.05 was considered statistically significant for all tests except for the heterogeneity test, in which case a *p* < 0.10 was used.

## 3 RESULTS

### 3.1 Characteristics of included studies

The PRISMA statement flowchart illustrated in Figure 1. shows the process of study selection. Of the 1639 records initially identified from the database search, 271 were duplicates filtered by software. Of the 1340 remaining articles, 1306 were inconsistent with the inclusion criteria and exclusion criteria by manual screening. In addition, full texts were not obtained for 3 articles. Then the remaining 31 records^19^, ^20^, ^21^, ^22^, ^23^, ^24^, ^25^, ^26^, ^27^, ^28^, ^29^, ^30^, ^31^, ^32^, ^33^, ^34^, ^35^, ^36^, ^37^, ^38^, ^39^, ^40^, ^41^, ^42^, ^43^, ^44^, ^45^, ^46^, ^47^, ^48^, ^49^ were reviewed. The detailed full‐article review resulted in 32 trials that met our inclusion criteria and were thus included in the present systematic review and meta‐analysis. The summarized characteristics of the 32 RCTs are shown in Table 1. A total of 1552 patients were grouped into the autologous epidermal cell suspensions group (*n* = 914) and control group (*n* = 883). Of these, 245 patients were in within‐subject controlled trials that was both experimental and control.

**TABLE 1:** Summary of characteristics of the randomized controlled trials included.

Study (author/year)	Journal	Country	Study design	Types of skin lesions	Intervention (Experimental and Control)	Number of patients	Male (n)	Female (n)	Age (year)	Skin lesion area (cm^2^)	Follow up
Gravante/2007	*Burns*	Italy	single‐center, prospective, RCT	Deep partial thickness burns	Recell	42	24	18	49 ± 9	186 ± 96	6 months
					Skin grafting	40	26	14	53 ± 10	180 ± 100	
Mulekar/2008	*British Journal of Dermatology*	Saudi Arabia	Single‐center, prospective, within‐subject controlled, RCT	Stable Vitiligo	Recell	5	3	2	23, 40, 40, 32, 18	Not mentioned	4 months
					Conventional melanocyte‐keratinocyte transplantation (MKT)						
Budania/2012	*British Journal of Dermatology*	India	Single‐center, prospective, RCT	Stable Vitiligo	Autologous non‐cultured epidermal cell suspension (NCES)	21	14	7	21 ± 4.087	Not mentioned	16 weeks
					Suction Blister Epidermal Grafting (SBEG)	20	12	8	21.3 ± 6.001		
Singh/2013	*British Journal of Dermatology*	India	Single‐center, prospective, RCT	Stable Vitiligo	Autologous non‐cultured epidermal cell suspension (NCES)	15	4	11	20.67 ± 4.761	18.8 ± 7.803	16 weeks
					Autologous non‐cultured outer root sheath hair follicle cell suspension (NCORSHFS)	15	6	9	23.33 ± 4.894	16.2 ± 15.497	
Liang/2013	*Nursing Research*	China	Single‐center, prospective, RCT	Wound bed preparation for sacrococcygeal massive pressure ulcers	Recell + Traditional dressing therapy + Negative pressure wound therapy	12	14	10	17 to 88	4 × 3.5 to 14 × 9	Not mentioned
					Traditional dressing therapy + Negative pressure wound therapy	12					
Liang/2014	*Modern Clinical Nursing*	China	Single‐center, prospective, RCT	Diabetic foot ulcers (DFU)	Recell	20	13	7	68.0 ± 3.6	7.0 ± 3.5	Not mentioned
					Standard skin grafting	20	11	9	69.0 ± 3.8	7.5 ± 4.0	
Hu/2015‐1	*Chin J Biomed Eng*	China	Single‐center, prospective, RCT	The donor site of skin grafting healed	Recell + Silver Sulphadiazine lmpregnated Hydrocolloid Dressing	15	12	3	22.4 ± 21.7	49.4 ± 42.8	12 weeks
					Silver Sulphadiazine lmpregnated Hydrocolloid Dressing	15	9	6	27.6 ± 22.5	54.1 ± 33.0	
Hu/2015‐2	*British Journal of Surgery*	China	Single‐center, prospective, double‐blinded, RCT	Chronic wound	Recell + Split‐thickness skin grafting (STSG)	44	30	14	48 ± 20	65 ± 48.3	7 months
					Split‐thickness skin grafting (STSG)	44	28	16	44 ± 18	58.8 ± 47.2	
Donaparthi/2016	*Indian Journal of Dermatology*	India	Single‐center, prospective, within‐subject controlled, open‐label, RCT	Stable Vitiligo	Epidermal melanocyte transfer (EMT)	6	1	5	18 ± 3.52	Not mentioned	6 months
					Hair follicular melanocyte transfer (HFMT)	5	4	1	31.4 ± 12.46		
Liu/2016	*Journal of Regional Anatomy and Operative Surgery*	China	Single‐center, prospective, RCT	Cicatricial hypopigmentation after deep partial thickness burns	Recell (Autologous pigment cell transplantation)	16	19	15	20 to 43	1×1 to 7×16	3 months
					Suction Blister Epidermal Grafting (SBEG)	18					
Hu/2017	*British Journal of Surgery*	China	Single‐center, prospective, single‐blind, RCT	The donor site of STSG	Recell + hydrocolloid dressing (Urgotul®)	53	40	13	51.3 ± 18.1	60.5 ± 32.2	12 weeks
					Hydrocolloid dressing (Urgotul®)	53	36	17	47.8 ± 16.7	56.9 ± 28.4	
Holmes/2018	*Journal of Burn Care & Research*	United States	Multicenter, prospective, within‐patient controlled, RCT	Acute burn	Recell	83	70	13	39.5 ± 13.1	10.0 ± 4.5	1 year
					Reticulate skin grafting						
Holmes/2019	*Burns*	United States	Multi‐center, prospective, evaluator‐blinded,within‐subject controlled, RCT	Mixed‐depth burn	Recell	30	25	5	39.1 ± 15.8	2443.0 ± 1675.0	1 year
					Split‐thickness skin grafting (STSG)						
Chen/2020	*Aesthetic Plastic Surgery*	China	Single‐center, retrospective, RCT	Acne scars	ReCell + skin scrape	48	19	29	26.33 ± 5.35	Not mentioned	1 year
					Skin scrape	30	17	13	26.10 ± 3.18		
Dalla/2020 (1)	*International Journal of Dermatology*	India	Single‐center, prospective, within‐subject controlled, RCT	Stable Vitiligo	Autologous non‐cultured epidermal cell suspension (NCES)	30	8	22	27.4 ± 10.7	12.3 ± 10.5	24 weeks
					Suction Blister Epidermal Grafting (SBEG)					7.7 ± 4.8	
Dalla/2020 (2)	*International Journal of Dermatology*	India	Single‐center, prospective, within‐subject controlled, RCT	Stable Vitiligo	Autologous non‐cultured epidermal cell suspension (NCES)	30	8	22	11.3 ± 6.5	12.3 ± 10.5	24 weeks
					Mini punch grafting (MPG)					5.6 ± 2.9	
Gunaabalaji/2020	*International journal of dermatology*	India	Single‐center, prospective, within‐subject controlled, open‐label, RCT	Vitiligo	Epidermal cell suspension (ECS)	20	13	7	23.9 ± 6.13	9.68 ± 3.14	9 months
					Hair follicle cell suspension (HFCS)					9.24 ± 3.56	
Hayes/2020	*International Wound Journal*	UK, France	Multicenter, prospective, RCT	Venous leg ulcers	Autologous skin cell suspension (ASCS) + Compression therapy	26	20	6	64.2 ± 16.5	14.25 ± 11.94	14 weeks
					Compression therapy	26	17	9	75.2 ± 11.66	13.37 ± 10.91	
Zhu/2020	*World Latest Medicine Information (Electronic Version)*	China	Single‐center, prospective, RCT	Vitiligo	Recell + fractional laser	28	18	10	27.4 ± 3.9	Not mentioned	Not mentioned
					Fractional laser	28	17	11	28.5 ± 3.8		
Li/2020	*World Latest Medicine Information*	China	Single‐center, prospective, RCT	Vitiligo	Recell + Ultraviolet‐phototherapy	51	25	26	33.58 ± 2.28	Not mentioned	3 months
					Conventional drugs (oral vitiligo pills combined with topical methoxalene solution)	51	26	25	34.15 ± 2.42		
Shi/2020	*Chinese Journal of Tissue Engineering Research*	China	Single‐center, prospective, RCT	Deep second‐degree burn	Recell	40	28	12	40.1 ± 8.31	Not mentioned	12 days
					Antibiotic Ointment	40	25	15	36 92 ± 9 32		
Hu/2020	*Chin J Biomed Eng*	China	Single‐center, prospective, RCT	Skin grafting area after tangential excision for deep partial thickness burns	Recell + Standard skin grafting	27	19	8	34.0 ± 9.4	171 ± 40	6 months
					Standard skin grafting	27	20	7	35.9 ± 12.1	193 ± 60	
Manning/2021	*Journal of Foot and Ankle Research*	Australia	Double‐center, prospective, open‐label, RCT	Diabetic foot ulcers (DFU)	Recell	24	19	5	61.5 ± 14.3	11 (8.4 to 15.3)	1 year
					Standard Care (SC)	25	21	4	58.1 ± 12.5	15.3 (9 to 23.8)	
Barnett/2021	*Journal of Hand Surgery Global Online*	United States	Single‐center, retrospective, RCT	Hand burns	Autologous skin cell suspension (ASCS) + Split‐thickness skin grafting (STSG)	37	26	11	41 ± 17	22 ± 14	1 year
					Split‐thickness skin grafting (STSG)	22	17	5	37 ± 12	8 ± 6	
Jesús/2022	*Journal of Clinical Medicine*	Mexico	Single‐center, prospective, RCT	Diabetic foot ulcers (DFU)	Cryopreserved allograft of human epidermal keratinocytes (Epifast)	40	22	18	65 ± 11.8	11.1 (2.5 to 32.5)	1 year
					Standard Care (SC)	40	19	21	63.1 ± 11.3	12.2 (3 to 28)	
Rao/2022	*J Cutan Aesthet Surg*	India	Single‐center, prospective, open‐label, RCT	Stable Vitiligo	Autologous non‐cultured epidermal cell suspension (NCES)	15	9	6	31.3 ± 13.55	1×2 to 7×8	4 months
					Smash skin grafting (SSG)	15	9	6	30.2 ± 11.05		
Singh/2022	*Indian Journal of Dermatology*	India	Single‐center, prospective, RCT	Chronic Non Healing Ulcer	Autologous noncultured epidermal cell suspension (NCES)	16	11	5	38.31 ± 9.76	222.94 ± 129.60	5 months
					Platelet‐rich fibrin matrix (PRFM)	19	13	6	41.68 ± 7.44	193.05 ± 165.11	
Dheemant/2023	*J Cutan Aesthet Surg*	India	Single‐center, prospective, open‐label, RCT	Chronic Nonhealing Trophic Ulcers in Patients with Hansen's Disease	Autologous Smashed Follicular Dermal Graft and Epidermal Cell Suspension	23	Not mentioned	Not mentioned	18 to 60	less than or equal to 5×5	6 months or complete healing
					Normal Saline Dressing	23					
Oberoi/2023	*Indian Journal of Dermatology, Venereology and Leprology*	India	Single‐center, prospective, within‐subject controlled, single‐blinded, RCT	Stable Vitiligo	Non‐cultured epidermal cell suspension (NCES)	30	11	19	≤ 20, 5 cases, 21−30, 9 cases, 31−40, 9 cases, > 40, 7 cases	Not mentioned	1 year
					Automated epidermal harvestingsystem (AEHS)						
Wang/2023	*Chinese Journal of Injury Repair and Wound Healing*	China	Single‐center, prospective, RCT	Deep second‐degree burn	Recell	20	12	8	38 ± 8.4	40 ± 1.54	1 year
					Reticulate skin grafting (1:2)	20	14	6	35 ± 7.2	43 ± 8.62	
Henry/2024	*Journal Of Trauma And Acute Care Surgery*	United States	Multicenter, prospective, within‐patient controlled, evaluator‐blinded, RCT	Full‐thickness skin defects	Autologous skin cell suspension (ASCS) + Split‐thickness skin grafting (STSG)	47	32	15	45.7	757.0 (778.9)	1 year
					Split‐thickness skin grafting (STSG)						
Chen/2024	*The Chinese Journal of Dermatovenereology*	China	Single‐center, prospective, RCT	Stable Vitiligo	Recell	30	13	17	23.2 ± 9.74	Not mentioned	2 years
					Suction Blister Epidermal Grafting (SBEG)	30	16	14	24.9 ± 12.96		

*Note*: Serial number definition:(1) ventilator‐associated pneumonia; (2) diarrhea; (3) clostridium difficile infection; (4) incidence of sepsis; (5) hospital acquired pneumonia; (6) length of mechanical ventilation days; (7) hospital mortality; (8) length of hospital stay; (9) ICU mortality rate; (10) length of ICU stay. The age data conforming to the normal distribution is expressed as M ± SD, the age data not conforming to the normal distribution is expressed as [M (P25, P75)].

Abbreviations: NA, not available; RCTs, randomized controlled trial.

These studies were published between 2007 and 2024, with sample sizes in individual trials ranging from 5 to 102. A total of 21 trials were published in English and 11 trials were published in Chinese. Eleven trials were carried out in the China, 10 trials in India, 4 trials in United States, 1 trial each in the Italy, Saudi Arabia, UK, France, Australia and Mexico. Almost all patients were adult participants.

Types of skin lesions in the included trials consisted of wound (including burn wound, skin grafting area after tangential excision or donor site after skin grafting), ulcers (including diabetic foot ulcers, venous leg ulcers and wound bed preparation for sacrococcygeal massive pressure ulcers), stable vitiligo, cicatricial hypopigmentation and acne scars. Interventions in the included trials consisted of different forms of NCES, and most of them were prepared using the ReCell technique. At the same time, there are new technologies, such as Cryopreserved allograft of human epidermal keratinocytes (Epifast) as an alternative to NCES, Alternatively, NCES could be combined with other treatments such as dressings, skin grafting, compression therapy or autologous Smashed Follicular Dermal Graft. Split‐thickness skin grafting was used in the control group. In the treatment of vitiligo, the control group also received conventional techniques such as conventional melanocyte‐keratinocyte transplantation (MKT) or suction blister epidermal grafting (SBEG). There are also new techniques such as autologous non‐cultured outer root sheath hair follicle cell suspension (NCORSHFS) or platelet‐rich fibrin matrix (PRFM). In wound repair related studies, in addition to the most common skin grafting, the control group in the trial also used traditional dressing therapy or NPWT. Except for the trials conducted by Liang in 2013 and 2014, which did not follow up patients after treatment, the rest of the trials were all followed up and the duration ranged from 12 days to 2 years. The detailed information of the included studies is summarized in Table 1.

### 3.2 Risk of bias and quality assessment of individual studies

**FIGURE 2:**
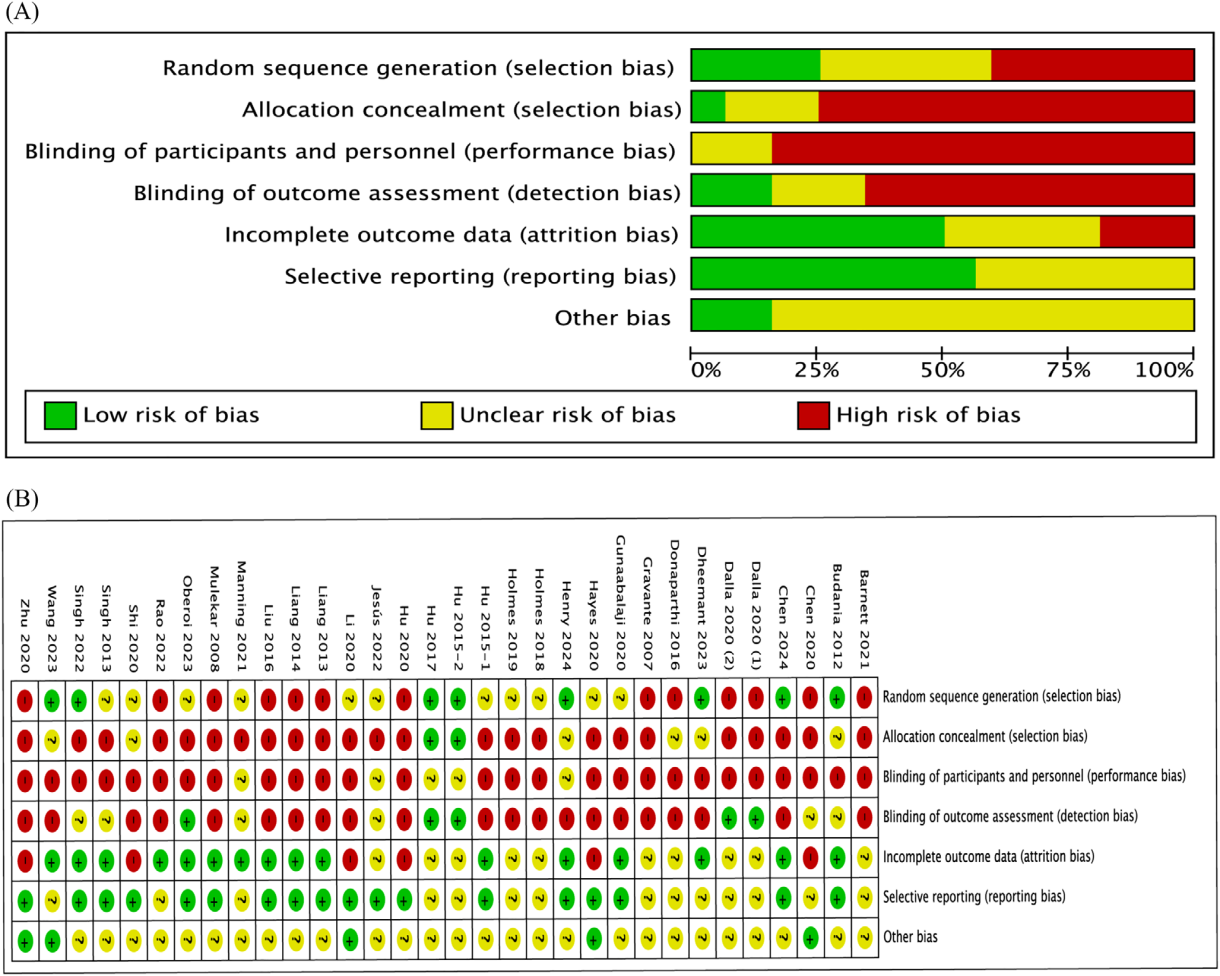
Risk of bias charts. (A) Risk of bias in the included studies; (B) Risk of bias summary for all included studies.

32 RCTs were assessed by the Cochrane Collaboration risk of bias tool. The characteristics of the included studies and their designs are shown in Figure 2 and Table 1, which summarizes the risk of bias of the included trials based on different quality domains of the risk of bias tool. Two studies were retrospective RCTs, the rest were prospective RCTs. In the 32 RCTs, the outcome assessors were double‐blinded only in one trial. The adequate randomized sequence generation was reported in 22% (7/32) of the trials but was unclear in 12 RCTs (12/32), and the remaining trials (12/32) used a method of randomization that included odd and even sampling by hospitalization number and clinician participation in allocation. For the all RCTs, one was double‐blinded, five were open‐label crossover trials, the rest are single‐blinded designs. All of RCTs, except 2 RCTs, had a high risk of bias in allocation concealment. While most of the RCTs had a high risk of bias in the blinding of participants and key study personnel, 2 RCTs had low risks of bias, and 2 RCTs had unclear risks of bias. In addition, all of the RCTs showed a relatively low risk of bias for incomplete outcome data and selective outcome reporting.

### 3.3 Meta‐analysis findings

#### 3.3.1 Healing time

**FIGURE 3:**
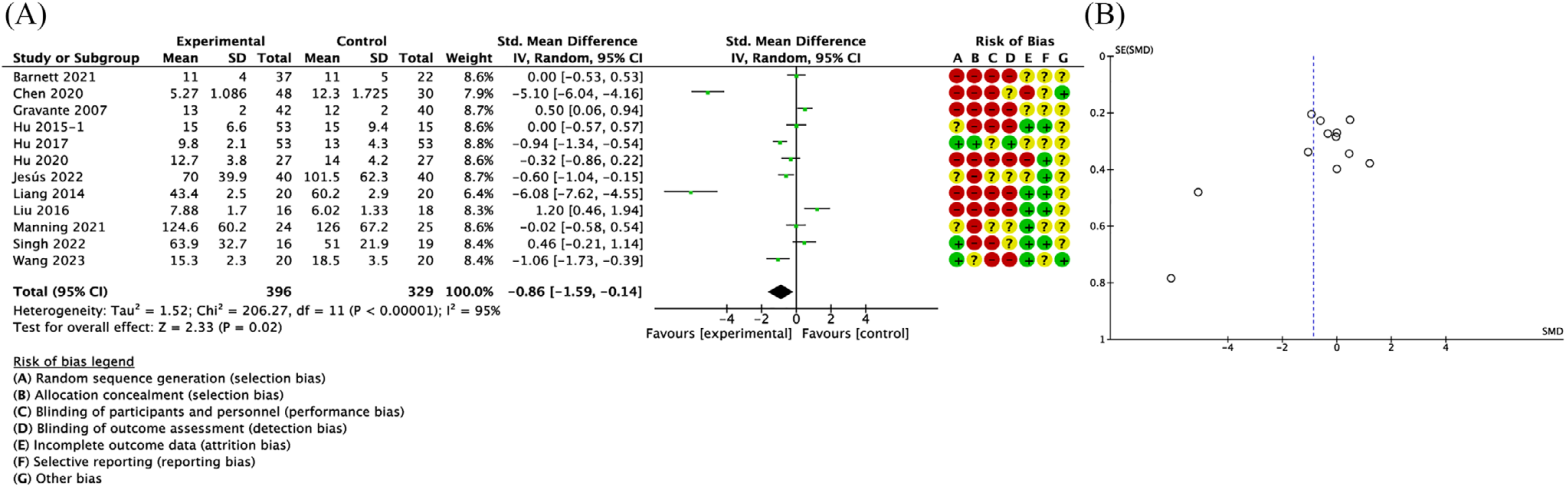
Forest plot and funnel plot of the meta‐analysis illustrating the overall weighted effect size of autologous epidermal cell suspensions versus control on the healing time. (A) Forest plot. The diamond symbol at the bottom of the forest plot represents the overall weighted estimate. Different colors (green, red, yellow) and symbols (“+”, “−”, “?”) to denote “low risk of bias”, “high risk of bias” and “unclear risk of bias”, respectively. (B) Funnel plot. The effect size “SMD” is shown on the abscissa, and the inverse of the standard error of the value of the effect size, SE (SMD), is shown on the ordinate. The dots in the figure are the individual studies included. SMD, standardized mean difference.

Twelve studies reported data on the healing time. Quantitative pooling of data revealed a significantly reduced healing time in patients receiving autologous epidermal cell suspensions compared with controls (SMD = −0.86; 95% CI: −1.59 to −0.14; *p* = 0.02, *I* ^2 ^= 95%) (Figure 3A). The funnel plot indicated no significant difference between the two groups (Figure 3B).

#### 3.3.2 Effective rate

**FIGURE 4:**
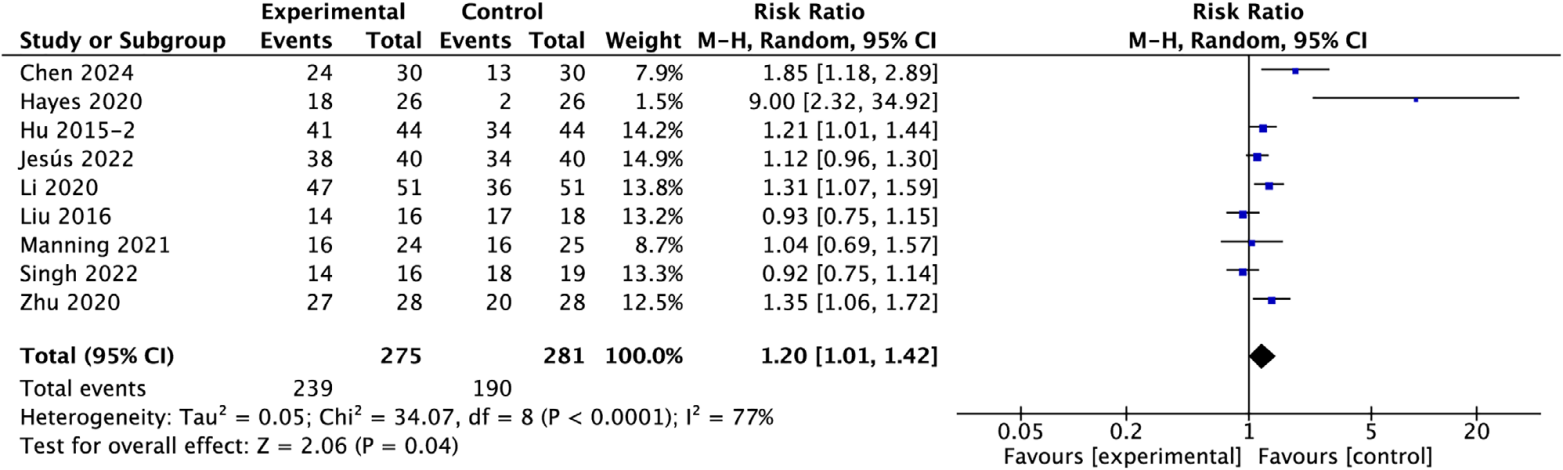
Forest plot of the meta‐analysis illustrating the overall weighted effect size of autologous epidermal cell suspensions versus control on the effective rate. The diamond symbol at the bottom of the forest plot represents the overall weighted estimate.

Complete data on the effective rate were available in nine studies. Pooled data from these trials demonstrated that there was a significant increasing in the effective rate of the experimental groups (RR = 1.20; 95% CI: 1.01–1.42; *p* = 0.04, *I* ^2 ^= 77%) (Figure 4). The funnel plot showed no evidence of publication bias (Figure S1).

#### 3.3.3 Size of donor site for treatment, size of study treatment area, operation time and pain scores

**FIGURE 5:**
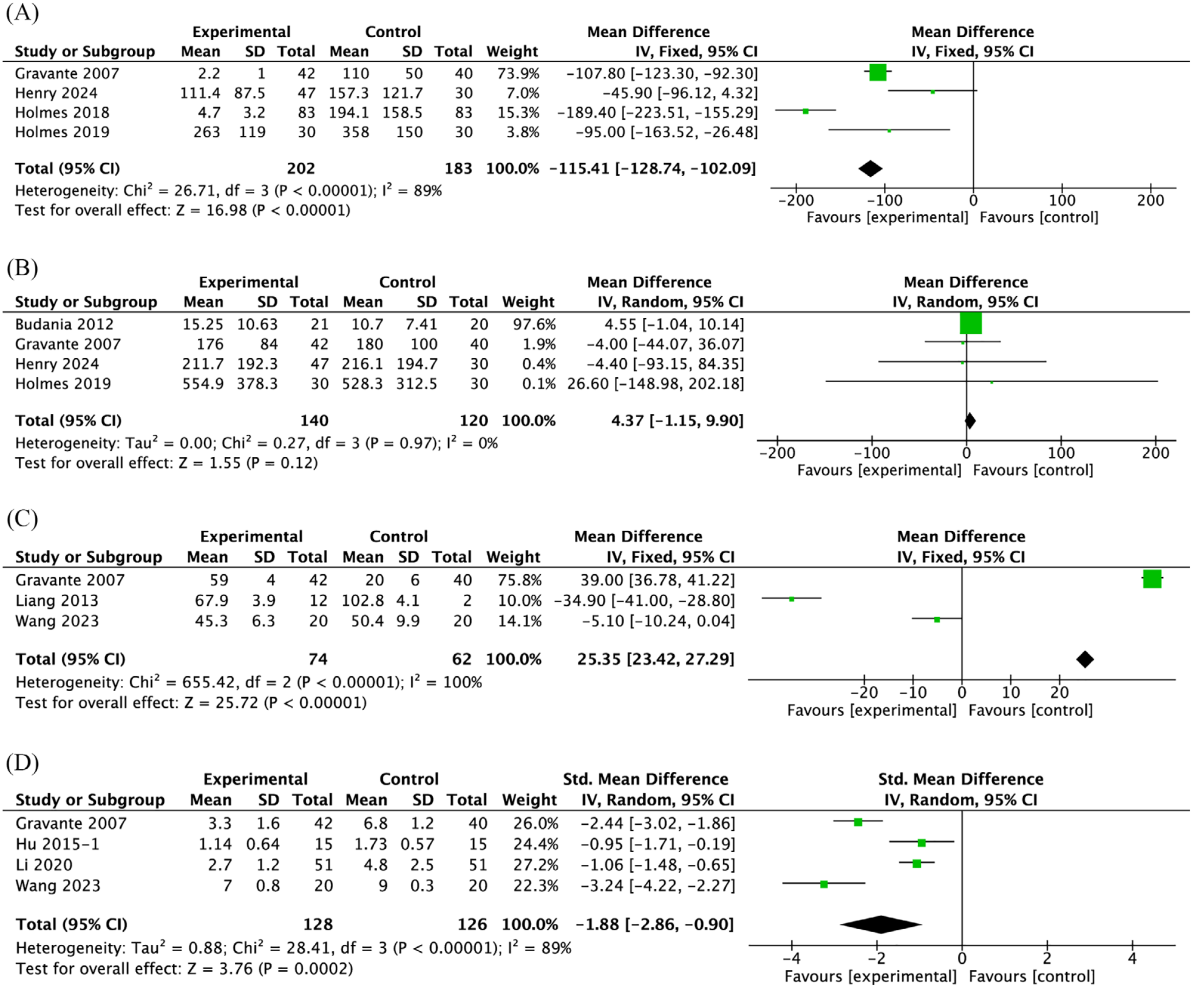
Forest plots of the meta‐analysis illustrating the overall weighted effect size of autologous epidermal cell suspensions versus control on the size of donor site for treatment, size of study treatment area, operation time and pain scores. (A) Size of donor site for treatment. (B) Size of study treatment area. (C) Operation time. (D) Pain scores.

The size of study treatment area was not significantly different between the treatment and control groups (MD = 4.37; 95% CI: −1.15–9.90, *p* = 0.12, *I* ^2^ = 0%) (Figure 5B), but the size of donor site for treatment was significantly lower in the treatment group than in the control group in four trials (MD = −115.41; 95% CI: −128.74 to −102.09; *p*<0.001, *I* ^2 ^= 89%) (Figure 5A). Autologous epidermal cell suspensions had significantly reduction on the operation time (MD = 25.35; 95% CI: 23.42–27.29; *p*<0.001, *I* ^2^ = 100%) and pain scores in RCTs (SMD = −1.88; 95% CI: −2.86 to −0.90; *p* = 0.0002, *I* ^2^ = 89%) (Figure 5C,D). All the funnel plots showed no publication bias in these analyses (Figure S2–S, 5).

#### 3.3.4 Repigmentation

**FIGURE 6:**
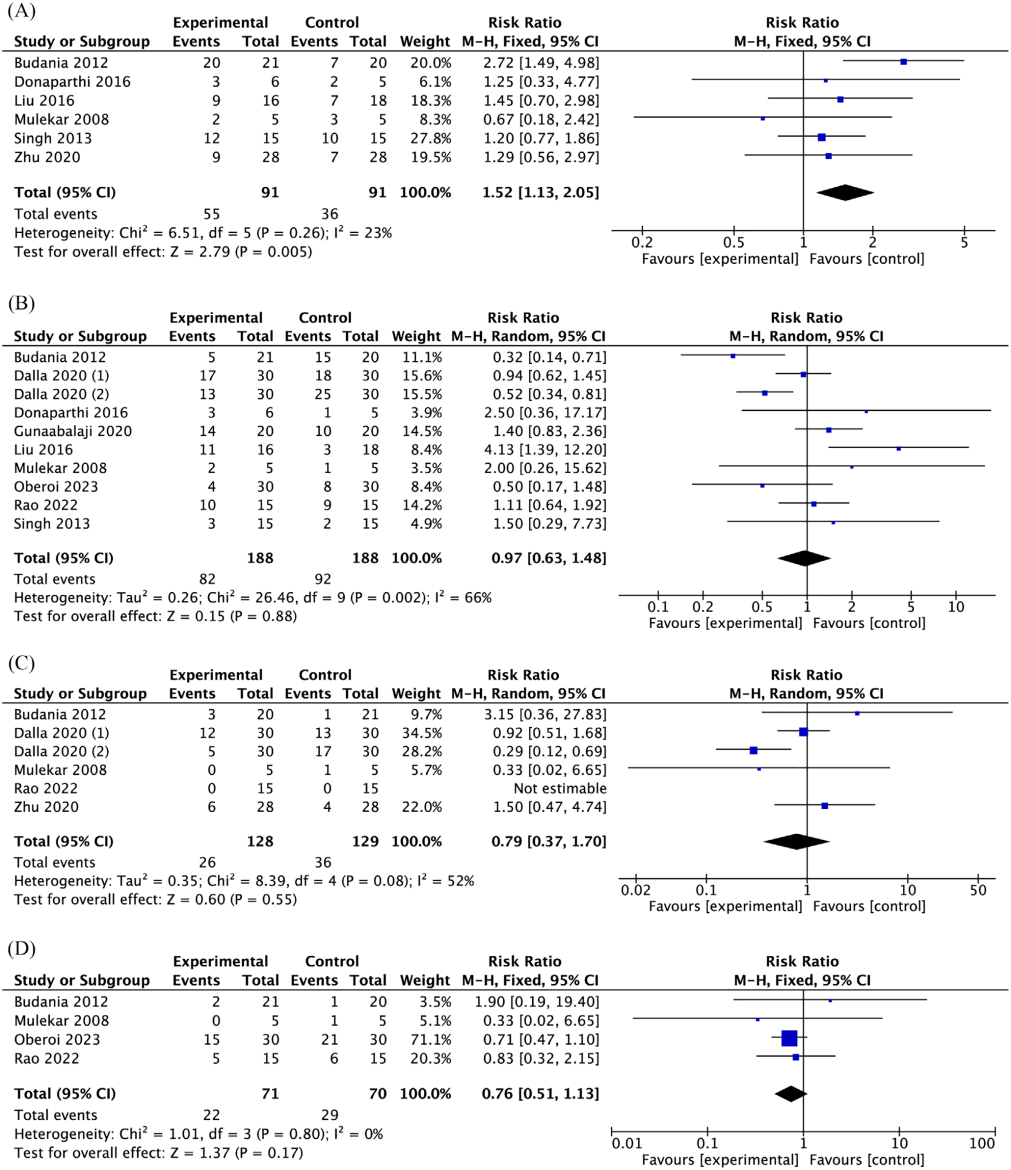
Forest plots of the meta‐analysis illustrating the overall weighted effect size of autologous epidermal cell suspensions versus control on the repigmentation. (A) 90%–100% repigmentation. (B) 75%–85% repigmentation. (C) 50%–74% repigmentation. (D) <50% repigmentation.

Meta‐analysis of 6 trials showed that the number of people who achieved 90%–100% repigmentation was more in the treatment group than in the control group (RR = 1.52; 95% CI: 1.13–2.05, *p* = 0.005, *I* ^2^ = 23%) (Figure 6A). However, there were no significant differences between the two groups in achieving 75%–85%, 50%–74%, and < 50% repigmentation (RR = 0.97, 0.79, 0.76; 95% CI: 0.63–1.48, 0.37–1,70, 0.51–1.13; *p* = 0.88, 0.55, 0.17; *I* ^2^ = 66%, 52%, 0%) (Figure 6B–D). The *I* ^2^ statistic indicated a high heterogeneity among two studies. However, funnel plot symmetry indicated that there was no detectable publication bias (Figure S6–S, 9).

#### 3.3.5 Complications

**FIGURE 7:**
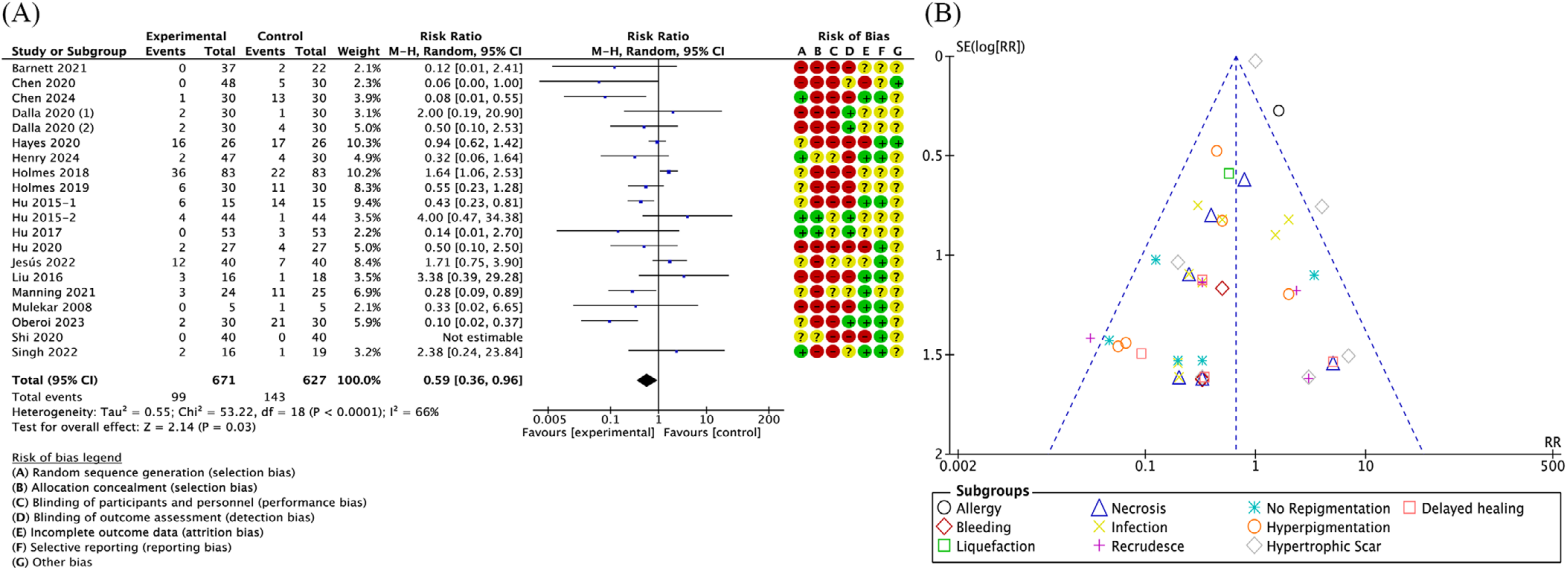
Forest plot and funnel plot of the meta‐analysis illustrating the overall weighted effect size of autologous epidermal cell suspensions versus control on the complications. (A) Forest plot. The diamond symbol at the bottom of the forest plot represents the overall weighted estimate. Different colors (green, red, yellow) and symbols (“+”, “–”, “?”) to denote “low risk of bias”, “high risk of bias” and “unclear risk of bias”, respectively. **(B)** Funnel plot. The effect size ”RR" is shown on the abscissa, and the inverse of the standard error of the value of the effect size, SE (log[RR]), is shown on the ordinate. The dots in the figure are the individual studies included.

Complications data was reported in 20 studies. There was a significant difference in complications between the treatment and control groups (RR = 0.59; 95% CI: 0.36–0.96, *p* = 0.03, *I* ^2^ = 66%) (Figure 7A). There was no significant publication bias in the selected studies, as indicated by funnel plot symmetry (Figure 7B).

**FIGURE 8:**
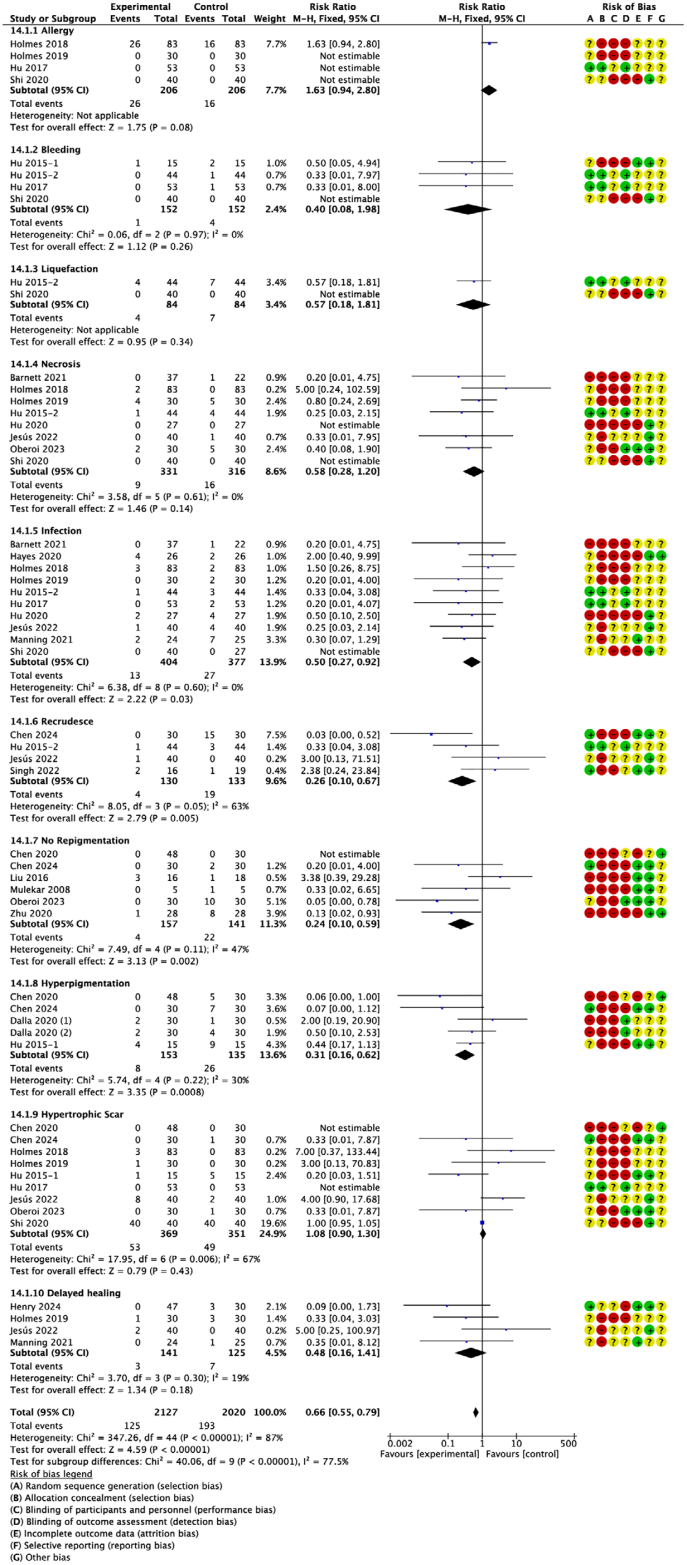
Forest plot of the subgroup analysis of the overall weighted effect size of autologous epidermal cell suspensions versus control on the different complications. The diamond symbol at the bottom of the forest plot represents the overall weighted estimate. Different colors (green, red, yellow) and symbols (“+”, “‐”, “?”) to denote “low risk of bias”, “high risk of bias” and “unclear risk of bias”, respectively.

Subgroup analysis also revealed that autologous epidermal cell suspensions could reduce the occurrence of infection, recrudesce, no repigmentation and hyperpigmentation (RR = 0.50, 0.26, 0.24, 0.31; 95% CI: 0.27–0.92, 0.10–0.67, 0.10–0.59, 0.16–0.62; *p* = 0.03, 0.005, 0.002, 0.0008; *I* ^2^ = 0%, 63%, 47%, 30%) (Figure 8), but autologous epidermal cell suspensions was not associated with a significant reduction in the allergy, bleeding, liquefaction, necrosis, hypertrophic scar and delayed healing.

#### 3.3.6 Scar scale scores

**FIGURE 9:**
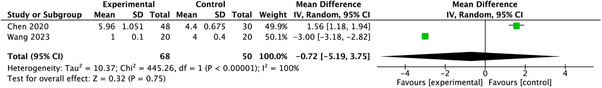
Forest plot of the meta‐analysis illustrating the overall weighted effect size of autologous epidermal cell suspensions versus control on the scar scale scores. The diamond symbol at the bottom of the forest plot represents the overall weighted estimate.

Scar scale scores was no significantly different between the treatment and control groups (MD = −0.72; 95% CI: −5.19–3.75, *p* = 0.75, *I* ^2^ = 100%) (Figure 9). The funnel plot was partially symmetrical, revealed no risk of publication bias (Figure S10).

#### 3.3.7 Satisfaction scores

**FIGURE 10:**
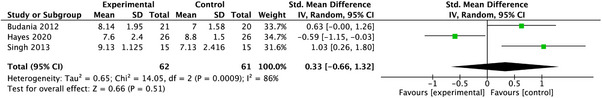
Forest plot of the meta‐analysis illustrating the overall weighted effect size of autologous epidermal cell suspensions versus control on the satisfaction scores. The diamond symbol at the bottom of the forest plot represents the overall weighted estimate.

The autologous epidermal cell suspensions among patients did not impacted on satisfaction scores as shown in the forest plots (SMD = 0.33; 95% CI: −0.66–1.32, *p* = 0.51, *I* ^2^ = 86%) (Figure 10), and the funnel plot did not show a biased pattern. (Figure S11.)

## 4 DISCUSSION

With the progression of daily life, production, transportation, as well as the development of radiation and chemotherapy, chronic illnesses, and an aging population, the skin being the outermost physical defense barrier of the human body, it is often the first to suffer damage. Common skin disorders like trauma, burns, pigment abnormalities, scars, and chronic ulcers pose significant physiological distress, mental strain, and economic burden to patients. One of the shared challenges among fields such as burn treatment, aesthetics, and reconstructive repair is restoring the normal form and functionality of damaged skin. Re‐epithelization of wounds is a crucial phase in healing processes. In cases of burns, trauma, and medically‐induced injuries, a deficit of skin islands on wound surfaces may prevent immediate healing. An optimal treatment strategy would consist of transplanting matching epidermal cells to the affected area and fostering their growth within suitable environments to expedite the re‐epithelization process, improving wound repair quality, and averting scar formation. Considering the principle of skin injury healing, a variety of treatment methods have been employed in clinical settings, aiming to counter different types of skin injuries. These include autologous skin grafting for burn treatment, NPWT for addressing various types of acute and chronic ulcers, blister epidermal grafting for treating pigment anomalies (like vitiligo), or laser therapy used for scar treatment. These therapeutic approaches vary in effectiveness, and sometimes, a combination is required for skin damage repair. Current studies are exploring the optimal method to repair skin injuries. With advancements in medical technology, a novel technique—ASCSs has been extensively applied to burns, aesthetics, and plastic surgery areas for skin wound repair, yielding notable results. ASCS builds upon the existing autologous epidermal cell grafting method, employing particular technology for autologous skin cell collection, processing, and transplantation. This involves converting autologous skin cells into suspension containing various cell components like keratinocytes and melanocytes and spraying them onto wounds for repair. The ReCell technology extends this approach, preparing the necessary epidermal cell suspension for wound repair in vitro without the need for cell culture, falling under the Autologous Non‐Cultured Epidermal Cell Suspension (NCES) technique. Compared to traditional treatment methods like skin grafting, research found that autologous epidermal cell suspensions have several advantages including alleviation of post‐operative pain, reduction of post‐operative scars, shorter hospitalization duration, restoration of original skin color, and cost reduction, while requiring a smaller donor site area. Despite the increasing number of RCTs addressing the treatment effects of autologous epidermal cell suspensions on various skin damages, due to the insufficient number of studies and design flaws in RCTs, the efficacy and safety of autologous epidermal cell suspensions in skin damage repair are yet to be determined. To this end, this systematic review and meta‐analysis aims to assess the efficacy and safety of autologous epidermal cell suspensions for re‐epithelialization of skin lesions.

In this study, we used autologous epidermal cell suspensions, primarily for treating vitiligo—a skin depigmentation disease with unknown origins. This ailment significantly impacts patients’ physical appearance and psychological wellbeing.^56^ Despite initial therapeutic attempts, many vitiligo patients do not experience satisfactory repigmentation, necessitating surgical intervention as the next treatment phase. As per Bassiouny,^57^ successful autologous non‐cultured cell transplantation requires the lesion to be stable for at least 12 months. In our study, vitiligo patients who had been stable for over a year post‐treatment, received autologous epidermal cell suspensions. Besides minimizing the donor site area, this procedure improves recoloration compared to traditional surgical methods like split‐thickness skin grafting or negative pressure blister epidermal grafting. Chen^58^ found surgical intervention to be effective for treating stable vitiligo, with positive results from both noncultured and cultured epidermal cell suspensions. Studies^59^, ^60^ found that repigmentation after autologous epidermal cell suspensions remained stable in most treated patches. Dev et al.^61^ posited that the preparation of recipient sites using manual dermabrasion (MD) or electrofulguration‐assisted dermabrasion influenced repigmentation rates. In terms of trypsinization methods in implementing noncultured epidermal cellular suspension, Rasheed^62^ observed that warmer and room temperature trypsinization techniques yielded significantly higher viability and repigmentation compared to the cold technique. Two recent RCTs^63^, ^64^ tested a combination of noncultured dermal/follicular and epidermal cell suspensions for clinical repigmentation in vitiligo patients, suggesting that such interventions could also be performed during unstable vitiligo phases. As for clinical safety, no serious adverse reactions were recorded in autologous epidermal cell treatments for vitiligo, consistent with findings by Holla et al.^65^ Sahni et al.^66^ reported similar safety in children and adolescents with stable vitiligo. Therefore, autologous epidermal cell suspensions present a safe and effective treatment for stable vitiligo, potentially useful in early‐stage interventions.

Given the limited research on the application of autologous epidermal cell suspensions for non‐healing or slow‐healing wounds, our study made a detailed analysis of eight trials.^23^, ^24^, ^26^, ^36^, ^41^, ^43^, ^45^, ^48^ These trials predominantly focused on chronic ulcer treatments such as pressure ulcers and diabetic foot ulcers. Our observations revealed that Autologous epidermal cell suspensions played a crucial role in reducing the epithelialization time in skin lesions and enhancing patient satisfaction post‐healing. These observations were consistent with Rashid et al.’s prospective, single‐arm feasibility study.^67^ In this study, non‐cultured ASCSs were used to treat diabetic foot ulcers. It was found that for ulcers that were not subjected to a soft tissue infection post‐treatment, the re‐epithelialization was satisfactory or achieved ≥95%, with some ulcers showing exposed tendon healing. Interestingly, another non‐controlled study^68^ suggested that non‐cultured autologous cell suspensions could not only enhance the appearance of healed chronic ulcers but also alleviate postoperative pain without serious adverse reactions. Thus, autologous epidermal cell suspensions may provide the necessary regenerative tissue stimulation for expedited healing of chronic ulcers, including those unresponsive to traditional methods. These findings point to the potential of this therapeutic strategy in wound management.

Hartmann et al.^69^ concluded that transplantation of autologous keratinocytes suspended in fibrin was an effective therapeutic approach in treating chronic venous leg ulcers. In a related study, Heyes et al.^35^ incorporated ASCS with compression therapy to address venous leg ulcers. Over 14 weeks, ulcers in the experimental group revealed a higher mean percentage of re‐epithelialization compared to the group solely under compression therapy. Additionally, patients reported lower levels of pain and higher satisfaction scores. In summary, a handful of well‐structured randomized trials have provided an initial validating evidence for the clinical efficacy ASCS‐based therapies for chronic wounds in human subjects. However, owing to the limited research thus far, there is a pressing need for larger sample size RCTs to further investigate the most optimal ASCS‐based treatment strategy for chronic ulcers.

We documented and analyzed complications that occurred in the included studies. Generally, autologous epidermal cell suspensions are associated with fewer complications compared to traditional wound treatments. In our study, infections and graft necrosis were the most commonly reported adverse events. We observed a clear statistical difference in the occurrence of these complications between the two groups. This contrast could be attributed to the high plasticity of the cell suspension, its ability to maintain tissue balance, and a lower requirement for substantial blood supply to the wound site. Additionally, an adequate presence of keratinocytes in the suspension can simultaneously promote rapid vascularization and epithelialization. The abundant melanocytes in the cell suspension might have contributed to preventing the lack of re‐pigmentation post‐surgery. We also found that, when compared to standard treatments, autologous epidermal cell suspensions reduced the recipient sites’ hyperpigmentation. However, due to the small number of studies, we did not record data on some complications, like amputation rates following diabetic foot ulcer surgery.^24^, ^41^, ^43^ These severe complications often resulted from the patients’ conditions, rather than the intervention processes implemented in these studies. Finally, because the data types in the records did not meet the conditions for inclusion in the meta‐analysis, only two studies and three studies were included in the meta‐analysis of whether autologous epithelial cell suspensions improved the scale scores and satisfaction scores, and no significant statistical difference was observed between the two groups in these two aspects. They are accompanied by high heterogeneity, and the results are not reliable.

Autologous epidermal cell suspensions, particularly autologous non‐cultured epidermal cell suspensions, do not need a special laboratory for execution. Doctors can carry out the process in a shorter time span, and postoperative care entails only simple dressing changes and wound care. In all experiments included in our study, we rarely observed complications like infection and scar hyperplasia. The technique minimizes the size of the donor site required for treatment, considerably reducing patient discomfort. Hu et al.^25^, ^29^ achieved rapid epithelialization and appropriate pigment recovery at the donor site after an autologous cell suspension spray, reducing donor site complications. This suggests that the ReCell technique is a simple, safe, and effective treatment for vitiligo plaques, burn wounds and a range of other acute and chronic wounds. When compared to split‐thickness skin grafting, the ReCell technique improves appearance and patient satisfaction considerably. Therefore, autologous epidermal cell suspensions emerge as a promising area for enhancing patient care outcomes in diverse wound management scenarios.

To provide a balanced interpretation of our findings and inform future research in this area, it's important to acknowledge both the strengths and limitations of this study. One limitation is the small number of studies included, which may affect the generalizability of the results. Furthermore, potential publication bias may restrict the applicability of our findings. For instance, most studies were conducted in China and India, which might limit the applicability of our results to individuals of European descent. Several sources of bias stem from differences in the cell suspension's preparation and usage methods, differences in wound type, and the inability to implement optimal blind design. The varying methodological quality of the included studies also poses a significant challenge. To minimize the bias and boost the evidence's credibility regarding the use of autologous epidermal cell suspensions in managing and rehabilitating skin lesions, it's crucial to conduct high‐quality RCTs with transparent reporting. Another challenge is the diversity in intervention methods and outcome measurements across studies. Standardizing therapy using autologous epidermal cell suspensions, treatment protocols, and outcome assessments could enhance the comparability of studies and strengthen the reliability of future meta‐analyses.

## 5 CONCLUSIONS

Autologous epidermal cell suspensions play a beneficial role in re‐epithelialization of skin lesion and presents a novel approach to the management of acute or chronic wounds, and vitiligo. This systematic review and meta‐analysis supports the potential role of autologous epidermal cell suspensions in reducing the healing time, size of donar site for treatment, operation time, pain scores and complications, as well as increasing the effective rate in skin lesions.

## CONFLICT OF INTEREST STAEMENT

The authors declare that they have no competing interests.

## ETHICS APPROVAL AND CONSENT TO PARTICIPATE

Not applicable.

## CONSENT FOR PUBLICATION

Not applicable.

## Supporting information

Supporting Information

Supporting Information

Supporting Information

Supporting Information

Supporting Information

Supporting Information

Supporting Information

Supporting Information

Supporting Information

Supporting Information

Supporting Information

## Data Availability

Data sharing not applicable to this article as no datasets were generated or analysed during the current study.
