# Supplementary figures and images for: The efficacy and safety of autologous epidermal cell suspensions for re‐epithelialization of skin lesions: A systematic review and meta‐analysis of randomized trials
Source: Skin Res Technol. 2024 Jun 19;30(6):e13820. doi: 10.1111/srt.13820 (PMC11186709; doi:10.1111/srt.13820)

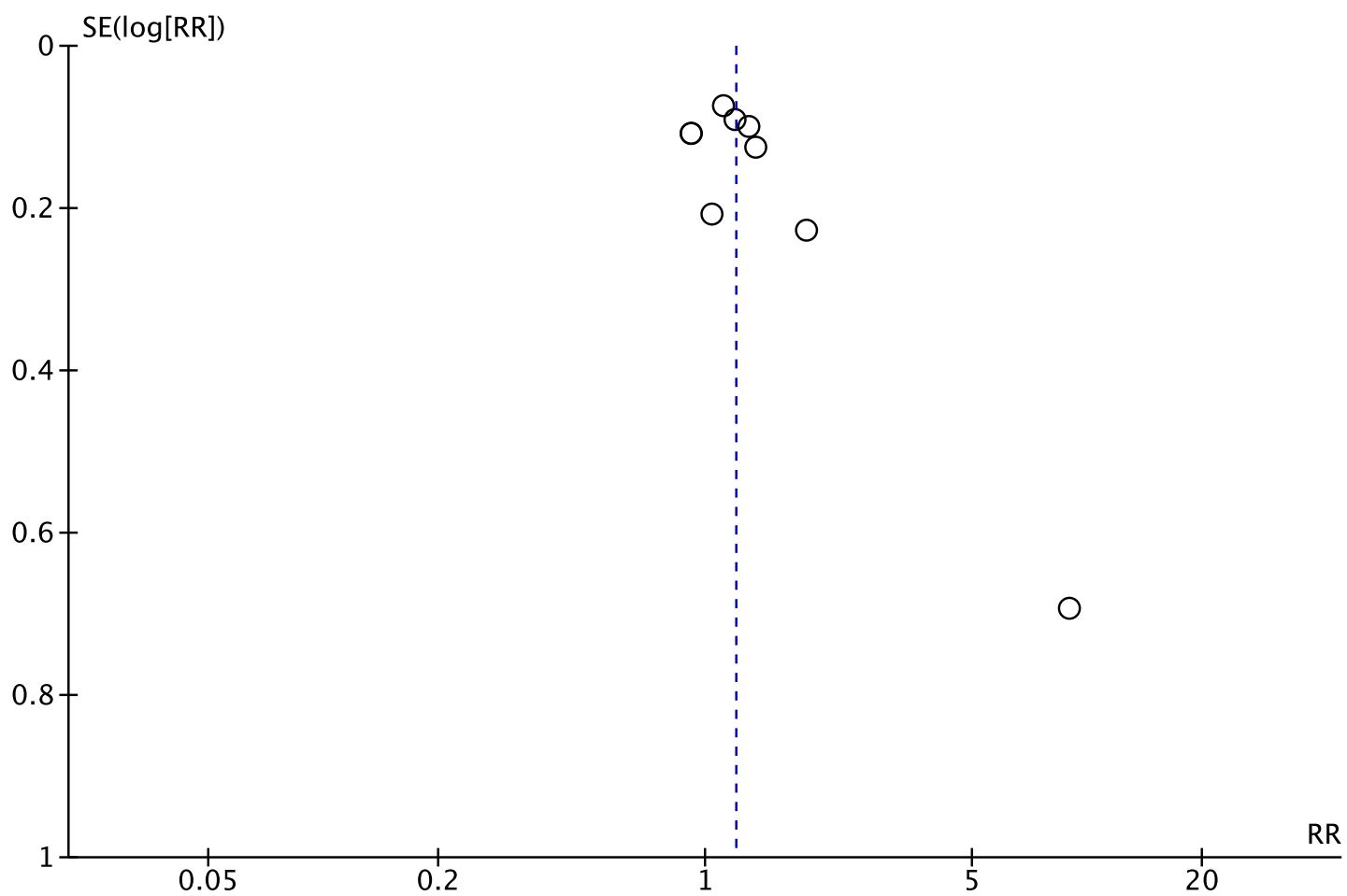

Supplement: Supplementary file 1 — Supporting Information [file SRT-30-e13820-s006.pdf]

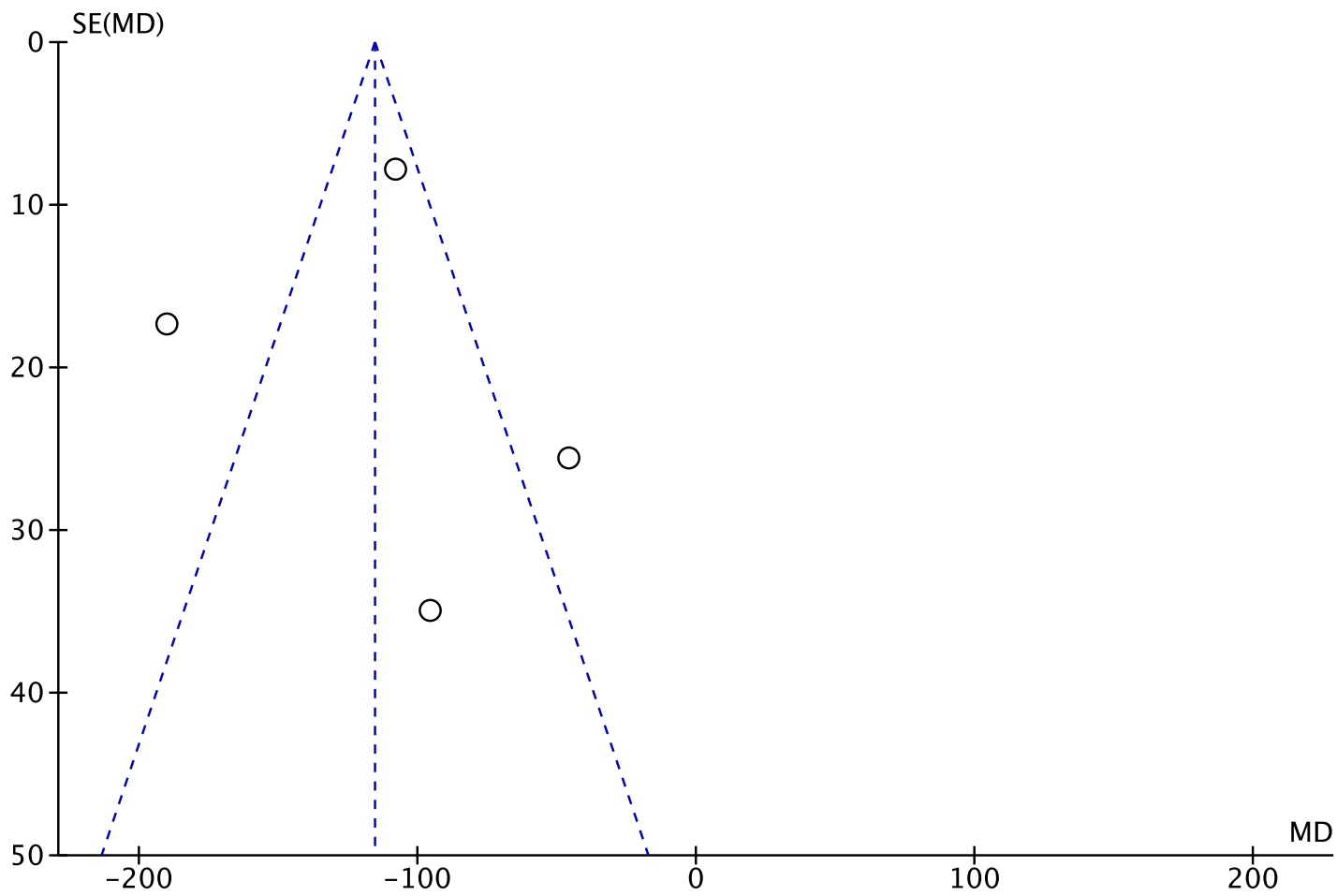

Supplement: Supplementary file 2 — Supporting Information [file SRT-30-e13820-s005.pdf]

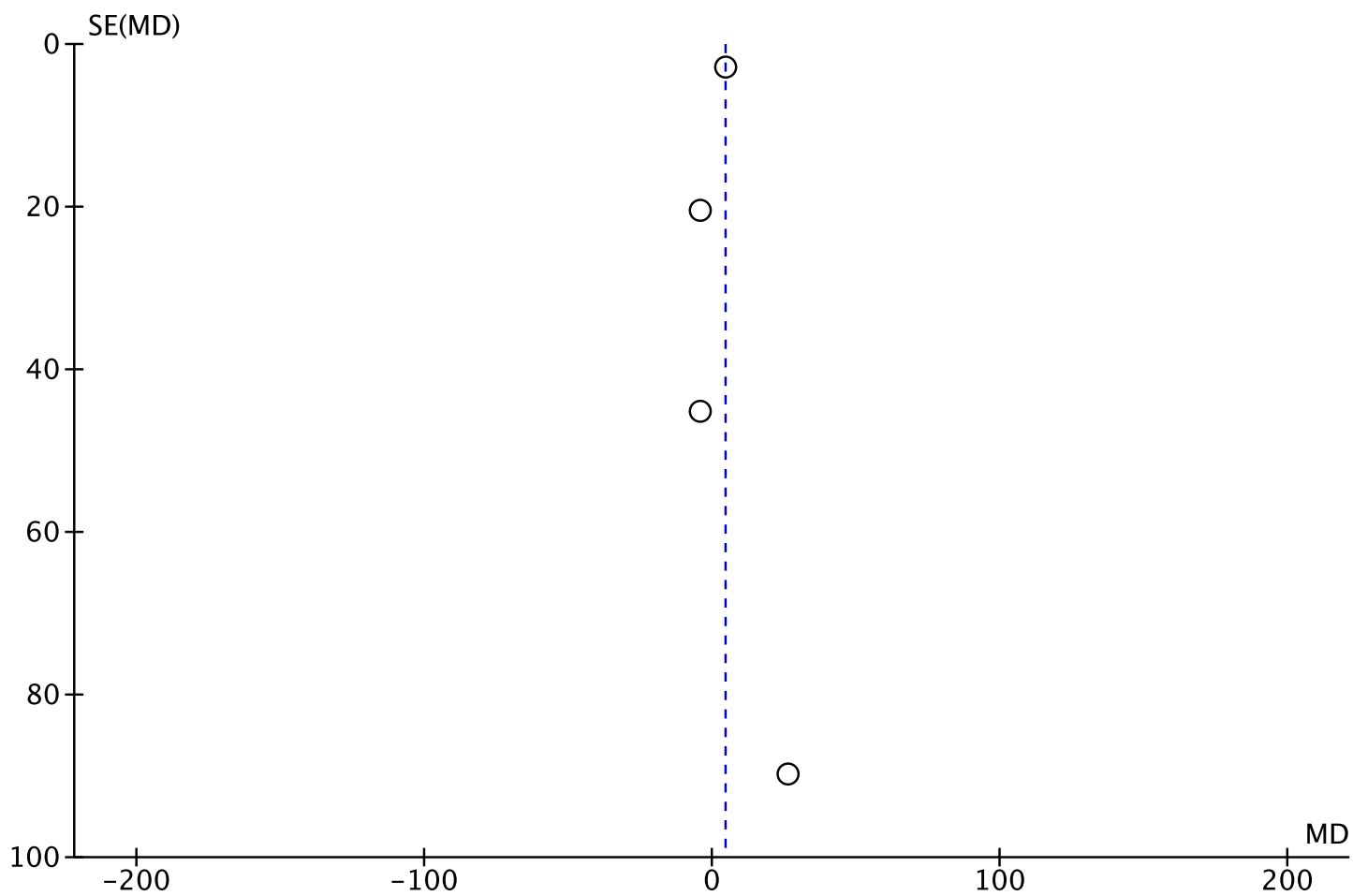

Supplement: Supplementary file 3 — Supporting Information [file SRT-30-e13820-s007.pdf]

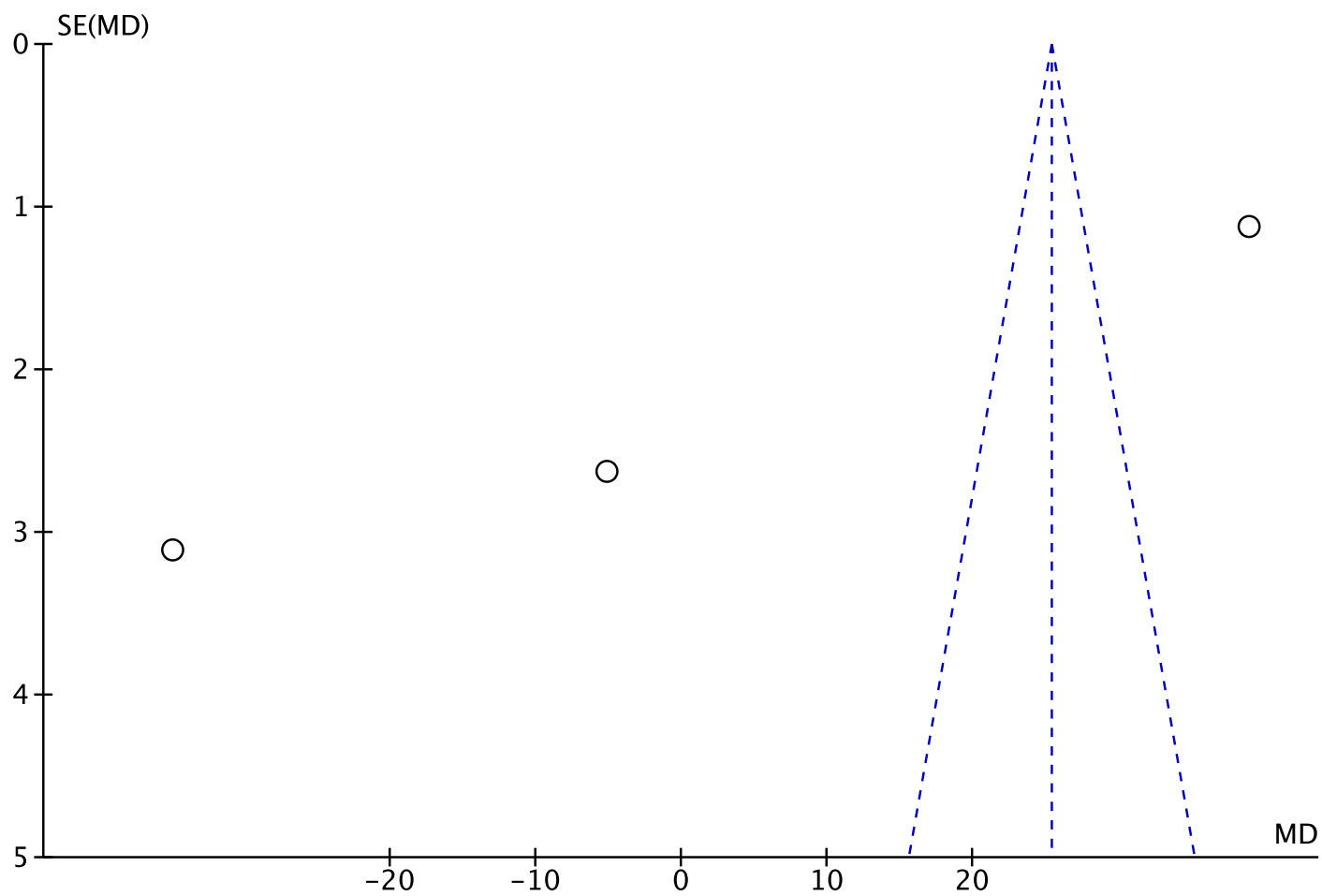

Supplement: Supplementary file 4 — Supporting Information [file SRT-30-e13820-s003.pdf]

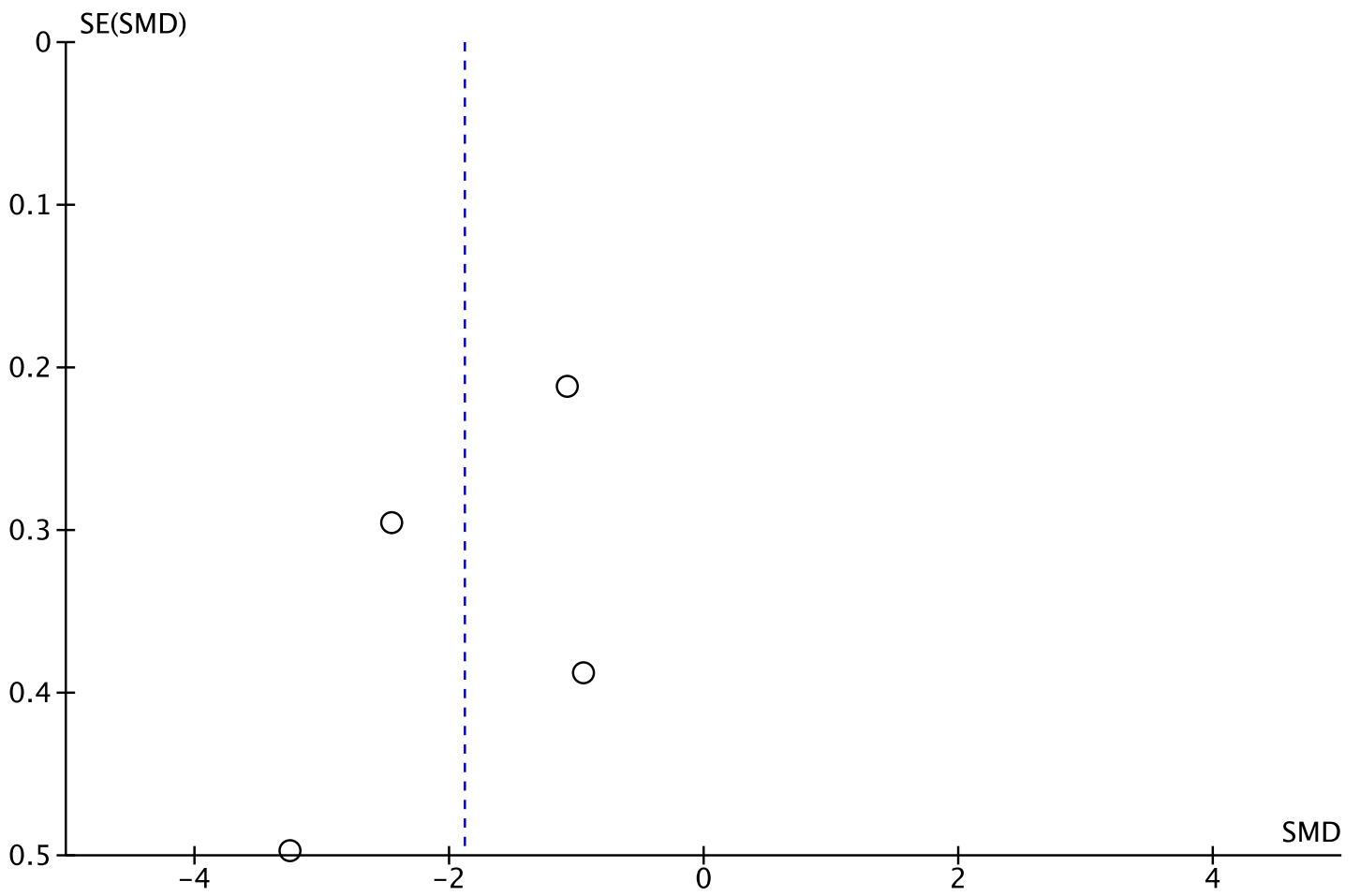

Supplement: Supplementary file 5 — Supporting Information [file SRT-30-e13820-s010.pdf]

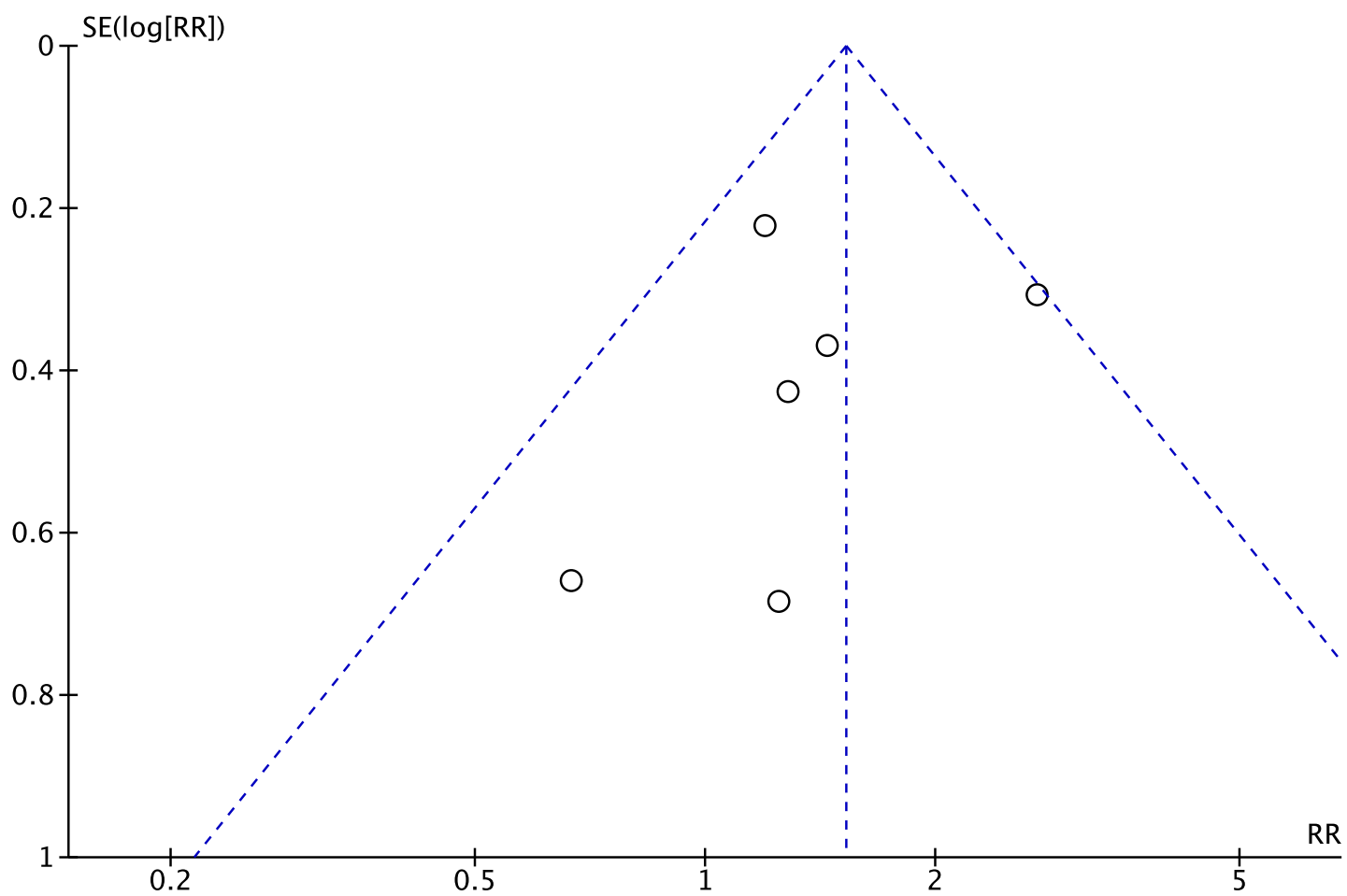

Supplement: Supplementary file 6 — Supporting Information [file SRT-30-e13820-s009.pdf]

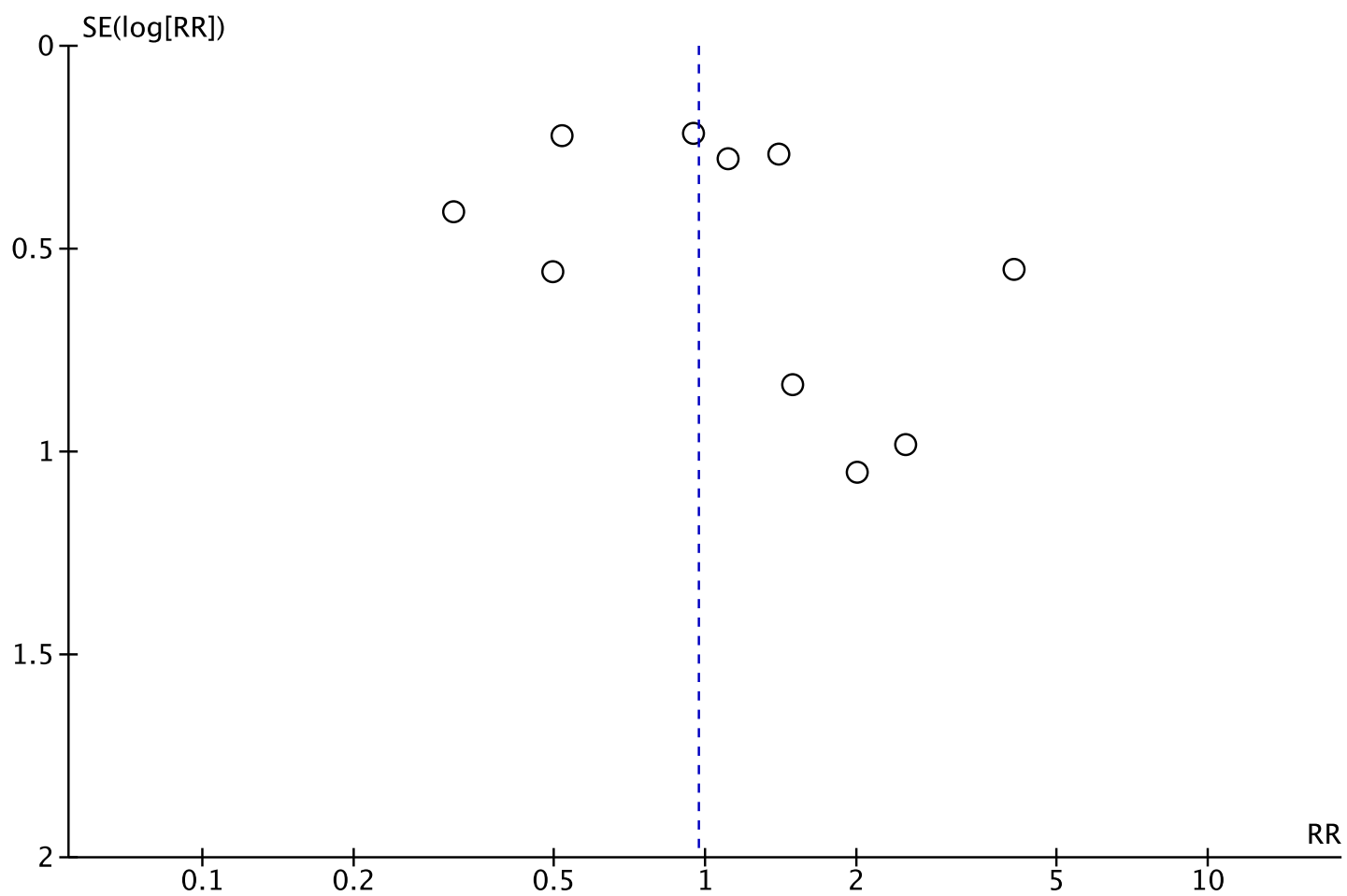

Supplement: Supplementary file 7 — Supporting Information [file SRT-30-e13820-s004.pdf]

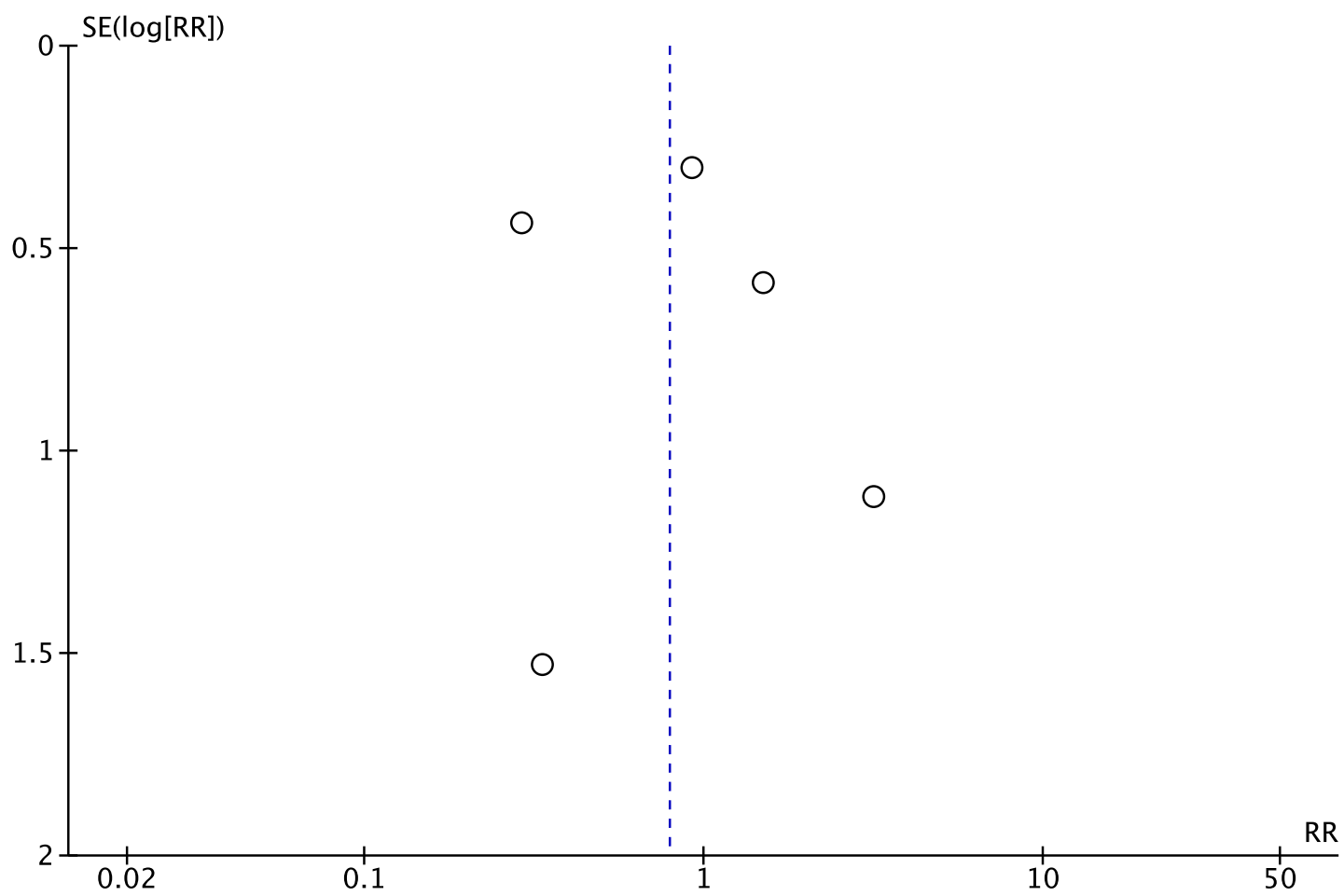

Supplement: Supplementary file 8 — Supporting Information [file SRT-30-e13820-s008.pdf]

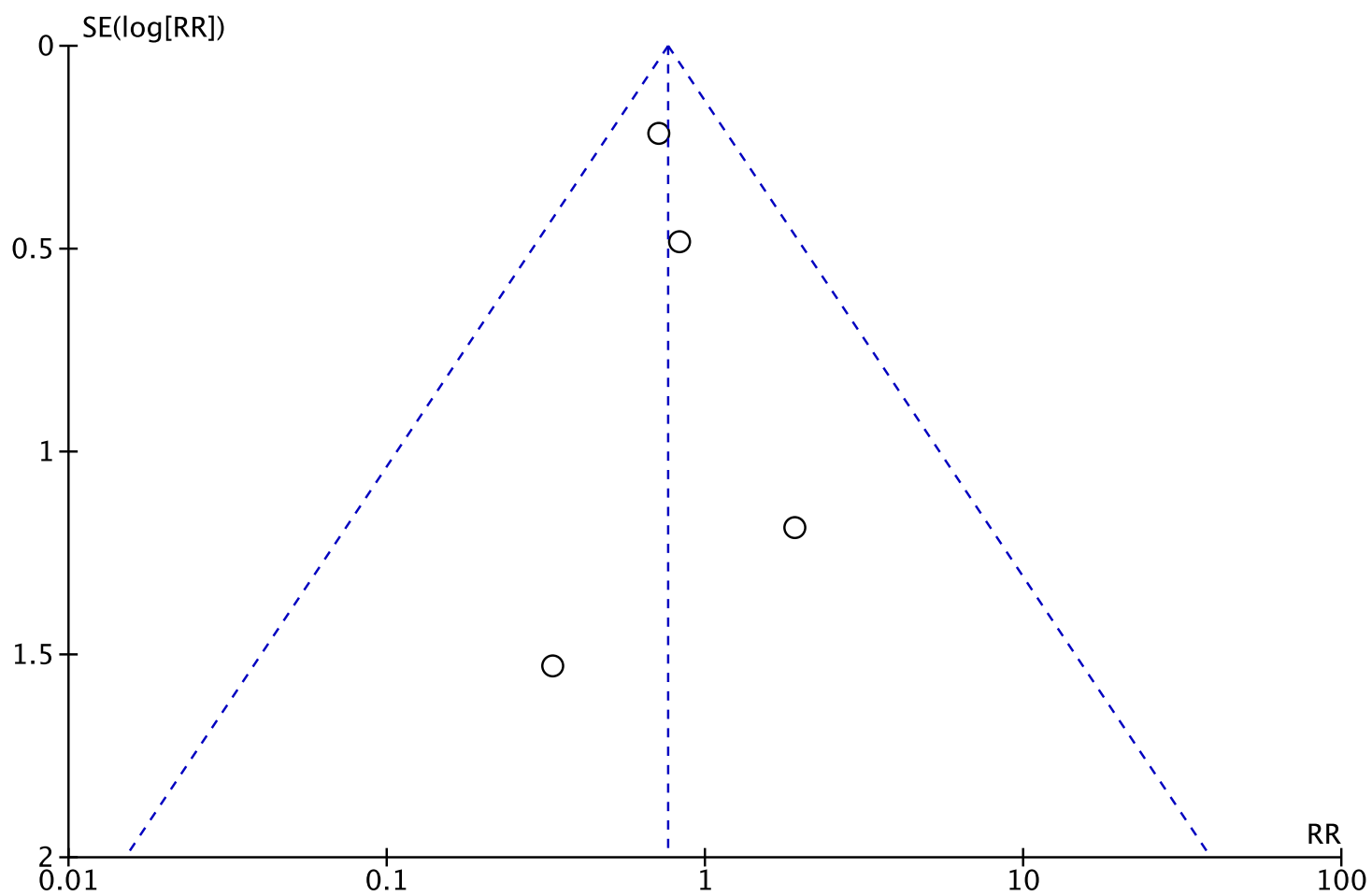

Supplement: Supplementary file 9 — Supporting Information [file SRT-30-e13820-s001.pdf]

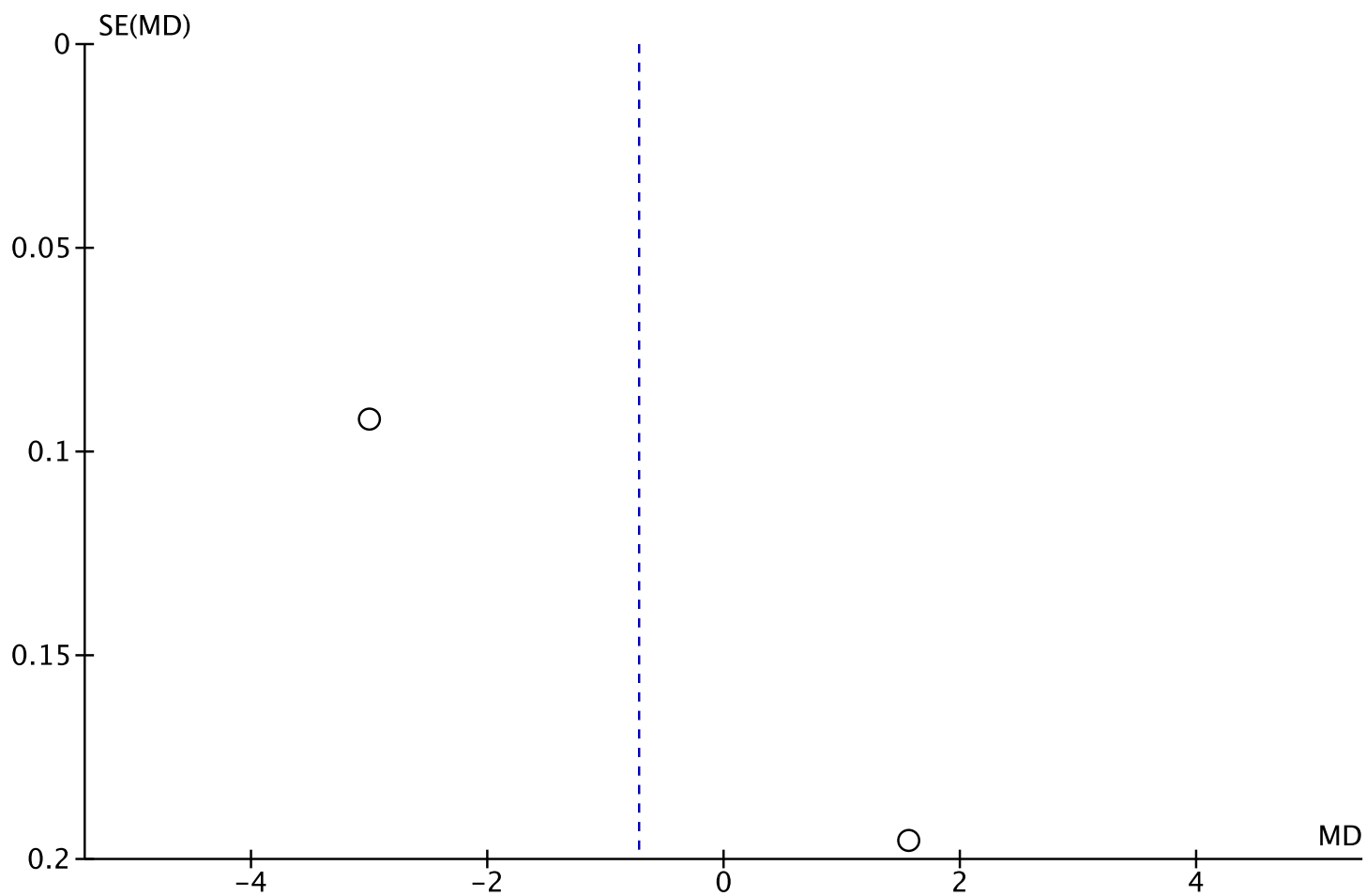

Supplement: Supplementary file 10 — Supporting Information [file SRT-30-e13820-s002.pdf]

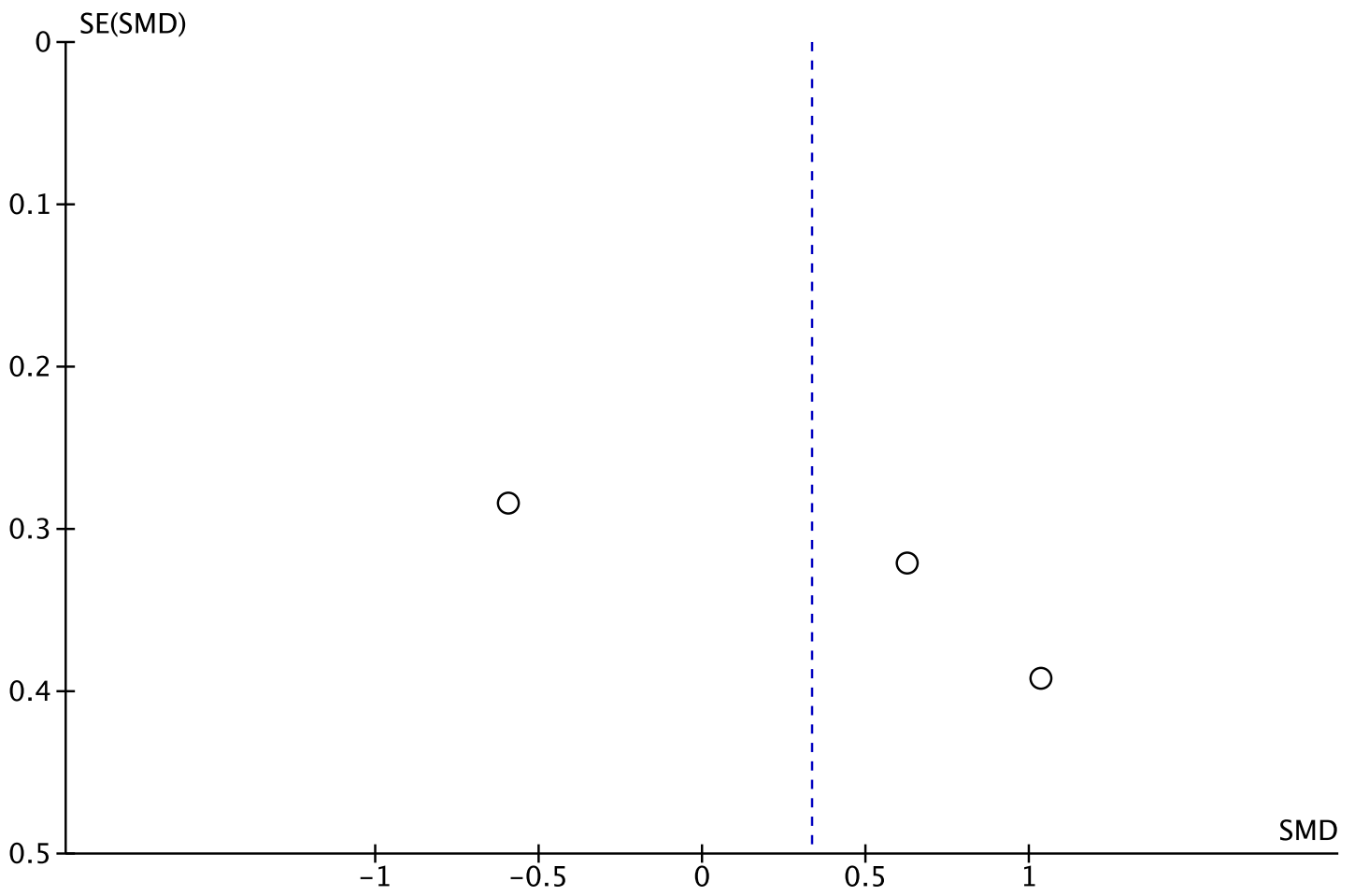

Supplement: Supplementary file 11 — Supporting Information [file SRT-30-e13820-s011.pdf]
